# Elevated expression of Gab1 promotes breast cancer metastasis by dissociating the PAR complex

**DOI:** 10.1186/s13046-019-1025-2

**Published:** 2019-01-21

**Authors:** Xiao Wang, Jing Peng, Ziqiang Yang, Pei-Jie Zhou, Na An, Lianzi Wei, Helen He Zhu, Jinsong Lu, Yu-Xiang Fang, Wei-Qiang Gao

**Affiliations:** 1 State Key Laboratory of Oncogenes and Related Genes, Renji-MedX Stem Cell Research Center, Ren Ji Hospital, School of Medicine, Shanghai Jiao Tong University, 160 Pujian Road, Shanghai, 200127 China; 2 School of Biomedical Engineering & Med-X Research Institute, Shanghai Jiao Tong University, Shanghai, 200030 China; 3 Department of Breast Surgery, Ren Ji Hospital, School of Medicine, Shanghai Jiao Tong University, 160 Pujian Road, Shanghai, 200127 China

**Keywords:** Breast cancer, Metastasis, Gab1, PAR complex, EMT

## Abstract

**Background:**

Breast cancer (BCa) remains as the second leading cause of cancer-related death in women worldwide. The majority of the deaths are due to its progression to metastatic BCa. Although Grb2-associated binding protein 1 (Gab1) has been implicated in tumor proliferation and metastasis in multiple tumors including colorectal cancer, hepatocellular carcinoma and ovarian cancer, whether and how it regulates BCa metastasis are still poorly understood.

**Methods:**

Western blot assay and immunohistochemical (IHC) staining were performed to assess expression of Gab1 in primary and metastatic BCa clinical samples. Biological function assay studies in vitro and in vivo were employed to investigate the functions of Gab1 during BCa metastasis. Co-immunoprecipitation (co-IP) assessment, western blot assay and immunofluorescence (IF) staining were carried out to investigate the underlying mechanism for the function of Gab1 on BCa metastasis.

**Results:**

In this study, we found that expression level of Gab1 was increased significantly in BCa tissue samples compared to that in benign mammary hyperplastic tissues. Furthermore, elevated expression of Gab1 was positively associated with metastasis in HER2 and TNBC subtypes of BCa. In BCa cell line MDA-MB-231 and SK-BR3 cells, stable overexpression of Gab1 promoted, while knockdown of Gab1 inhibited cell migration in vitro and metastasis in vivo. Mechanistically, overexpression of Gab1 enhanced its interaction with Par3, a key component of the polarity-associated partitioning defective (PAR) complex, leading to a dissociation of the PAR complex. Consequently, dissociated PAR complex induced epithelial-to-mesenchymal transition (EMT) for breast tumor metastasis. By restoration assessment, we found that only re-expression of a fully functional Gab1, but not a mutant Gab1 that harbors either Par3 binding-deficiency or Par1b binding-deficiency, could reverse the repressive phenotype of cell migration in vitro and metastasis in vivo due to Gab1 knockdown.

**Conclusions:**

Our findings indicate that elevated expression of Gab1 promotes BCa metastasis by dissociating the PAR complex that leads to EMT, implicating a role of Gab1 as a potential biomarker of metastatic BCa. Moreover, inhibition of Gab1 expression might be a promising therapeutic strategy for BCa metastasis.

**Electronic supplementary material:**

The online version of this article (10.1186/s13046-019-1025-2) contains supplementary material, which is available to authorized users.

## Background

Breast cancer (BCa) is the most frequently diagnosed disease in women worldwide. Compared to early-stage BCa patients, metastatic BCa patients have an extremely lower five-year survival rate (90% vs. 25%) [1, 2]. It is documented that about 6% of patients are found to have metastatic diseases during the first diagnosis, due to the limitation of effective early diagnostic biomarker of metastasis [3]. Moreover, in spite of the progress in therapy of metastatic BCa, underlying molecular mechanisms of BCa metastasis still need to be further explored.

Grb2-associated binding proteins (Gab), including Gab1, Gab2, and Gab3 in mammals, are well-known to work as scaffold proteins for protein-protein interaction and are regarded as signal ‘amplifiers’ in transduction of multiple signal pathways during tissue development [4, 5]. Recently, their involvement in tumor initiation and progression has been explored. For instance, overexpression of Gab2 is suggested to be tightly associated with tumor proliferation and metastasis in BCa, melanoma and ovarian cancer [6–8]. Besides Gab2, Gab1 is also shown to be positively correlated with tumor proliferation and metastasis in head and neck squamous cell carcinoma and colorectal cancer [9, 10]. However, the relationship between Gab1 expression level and BCa metastasis and the potential mechanism of Gab1 for regulation of BCa metastasis remain unclear. In particular, whether Gab1 can regulate BCa metastasis via a mechanism that is different from previous ones is unknown.

PAR complex, an evolutionarily conserved protein complex among multicellular organisms from worms to humans, is important for maintenance of epithelial polarity and its dislocation or dissociation is involved in epithelial tumorigenesis and metastasis [11, 12]. As reported, loss of Par3, a key component of the PAR complex, is found to be tightly correlated with lymph node metastasis in esophageal squamous cell carcinoma [13]. Our previous work also demonstrated that dissociation of PAR complex by Shp2 contributes to metastasis in prostate cancers via induction of epithelial-to-mesenchymal transition (EMT) [14]. Importantly, our other previous work provided evidences that Gab1 mediates an interaction of Par1b with Par3 to induce phosphorylation of Par3 by Par1b, resulting in a dissociation of PAR complex in epithelial cells [15]. Considering together, it is worth investigating whether Gab1 exerts its effect on regulation of BCa metastasis via influencing stability of the PAR complex.

In the present study, we aimed to determine possible roles of Gab1 in BCa metastasis. We found that the expressional level of Gab1 is significantly upregulated in patients with metastatic BCa. Mechanistically, we demonstrated a new mechanism by which Gab1 can dissociate the PAR complex to promote BCa metastasis by inducing EMT. Our findings suggest Gab1 as a potential diagnostic biomarker of BCa metastasis and also a promising target for anti-metastatic therapy in BCa.

## Methods

### Cell lines and cell culture

BCa cell lines MDA-MB-231 and SK-BR3 were purchased from the American Type Culture Collection (ATCC, Rockville, MD, USA). Cells were grown in Dulbecco’s modified Eagle’s medium (DMEM, Thermo Fisher Scientific, Waltham, MA, USA) plus 10% fetal bovine serum (FBS, Thermo Fisher Scientific) and maintained at 37 °C in a humidified atmosphere of 5% CO_2_.

### Plasmid construction

Gab1 shRNA#1~#3 (three independent shRNA sequence) and scrambled shRNA control were designed and constructed into a lentiviral expression vector pGMLV-SC5 respectively by Genomeditech Comp. (Shanghai, China). Lentiviral expression vectors for expression of a synonymous mutation of Gab1 (NM_207123.2, Gene ID: 2549) for antagonist of Gab1 shRNAs (herein named Gab1WT, 115KDa), a Par1b binding domain (152-250aa) or Par3 binding domain (301-400aa) deletion mutant of Gab1 (named Gab1MT1, 103KDa, or Gab1MT2, 103KDa) and related vector control were constructed by Genomeditech Comp.

### Lentivirus production and infection

All above mentioned lentiviral expression vectors were packaged into lentivirus respectively by Genomeditech Comp. (Shanghai, China). For infection, 1.5 × 10^5^ cells were cultured in 6-well plate, and relevant lentiviral vectors (MOI = 10 for each) were added along with 5 μg/ml polybrene (final concentration) into the cultures. Culture supernatant was replaced by fresh medium containing 10% FBS 24 h after infection. Expression of Gab1 was measured by western bolt. MOI: multiplicity of infection.

### Transwell assay and wound healing assessment

For transwell assay, 3 × 10^4^ MDA-MB-231 cells or 5 × 10^4^ SK-BR3 cells were seeded in the upper chamber (Corning, NY, USA) with 400 μl DMEM, while the lower chamber was loaded with 800 μl DMEM containing 10% FBS. After incubation for 24 h, transmigrated cells (on the lower surface of membrane) were fixed with 4% paraformaldehyde, consequentially stained with 1% crystal violet for 15mins at room temperature, photographed and counted under a microscope in five random fields.

For wound healing assay, 6 × 10^5^ cells were seeded on the 6-well plate. After starvation in serum-free medium for 24 h, the linear wound was made using a sterile 200 μl tip. Respective images were observed and photographed under the microscope at 0 h and 24 h after wound production respectively.

### In vivo tumor metastasis assay

Six-week-old female BALB/C athymic nude mice (SLAC, Shanghai, China) were housed and manipulated according to the protocols approved by the Renji Hospital Medical Experimental Animal Care Commission. To establish a lung metastasis model,1× 10^6^ cells were injected intravenously. Four weeks later, mice were sacrificed and lungs were collected and fixed in 4% formaldehyde for H&E and immunofluorescent (IF) staining respectively. The number of metastatic nodes in lung tissues was counted in five mice in each group.

### Clinical samples

Investigation has been conducted in accordance with the ethical standards and according to the Declaration of Helsinki and national and international guidelines. BCa samples for western blot assay and immunohistochemical (IHC) staining, paired adjacent normal tissue samples for western blot assay and non-paired benign mammary hyperplastic samples for IHC staining were obtained from Department of Breast Surgery, Ren Ji Hospital (Shanghai, China). Written informed consents were obtained from all patients for the application of the specimens used in the research.

### Immunofluorescent (IF) staining

Cells were seeded on cover slides placed in 24-well plate and cultured in DMEM medium supplemented with 10% FBS and maintained at 5% CO_2_ at 37 °C for 48 h. Adherent cells on cover slides were fixed with 4% paraformaldehyde for 20mins at room temperature. Cells were permeabilized with 0.3% Triton X-100 and then blocked with 10% normal donkey serum (GeneTex, Irvine, California, USA) for 30mins at room temperature for IF staining. Tissues were fixed in 4% paraformaldehyde overnight and embedded in paraffin. Paraffin-embedded tissue sections were performed by Runnerbio biotech. Comp. (Shanghai, China) and were dewaxed in xylene for 5mins and then hydrated in 100, 95, 85 and 70% ethanol successively. After inactivating endogenous peroxidase in disodium-hydrogen phosphate-2-hydrate, these sections were blocked in 10% donkey serum for 1 h at room temperature for IF or IHC staining (see below). For IF staining, after incubated with relevant primary antibody (1:200, diluted in PBS with 1% normal donkey serum) at 4 °C overnight, cells or sections were first washed with PBS buffer three times (10mins each) and in turn incubated in dark with Alexa Fluor-594 or Alexa Fluor-488 conjugated secondary antibody (Thermo Fisher Scientific) at room temperature for 1 h. Cells and sections were washed with PBS three times again before mounted with DAPI (Thermo Fisher Scientific). Images of IF staining were observed and photographed by a microscope (Leica DFC420C). After co-staining for Gab1 and EpCAM in human tumor tissue samples, the relative expression of Gab1 was evaluated by the following formula: relative intensity ratio of Gab1 expression = intensity of Gab1 staining / intensity of EpCAM staining. The effectiveness and specificity of primary antibodies were checked before IF staining by no-primary antibody control assay. All antibodies are available in Additional file 1: Table S1.

### H&E staining and Immunohistochemical (IHC) staining

H&E staining for paraffin-embedded tissue sections was carried out by Runnerbio biotech. Comp. (Shanghai, China). For IHC staining, sections were incubated with Gab1 antibody (1:200) at 4 °C overnight. After washed with PBS for three times (10 min each), sections were incubated with horseradish peroxidase-conjugated secondary antibody (Vector, Burlingame, CA, USA) for 1 h at room temperature. Sections were then washed with PBS again for three times and were visualized with DAB (Sangon Biotech, Shanghai, China) staining and hematoxylin counterstaining (Beyotime, Shanghai, China). Images were acquired under a microscope (Leica DFC420C) in the same exposure conditions. The effectiveness and specificity of primary antibodies were checked before IHC staining by no-primary antibody control assay. All antibodies are available in Additional file 1: Table S1.

### Western blot and co-immunoprecipitation (co-IP)

Harvested cells were rinsed twice in PBS, lysed in RIPA supplemented with protease inhibitor and PMSF (Thermo Fisher Scientific) for 30mins on ice. Clinical samples including the normal tissues and tumor tissues were firstly sonicated for 20s and then were lysed under the same condition. After measured by BCA (bicinchoninic acid) kit (Thermo Fisher Scientific), 30 μg of total proteins were separated by SDS-PAGE and transferred to PVDF membrane (Millipore, Bedford, MA, USA). The membrane was blocked with TBST containing 5% BSA at room temperature for 1 h and then probed with the relevant primary antibodies at 4 °C overnight, which was then replaced by an HRP-conjugated secondary antibody at room temperature for 1 h. The protein immunoreactive bands were visualized by the ECL detection instrument (Thermo Fisher Scientific) plus chemiluminescent substrate.

For co-IP, whole cell lysates were prepared using a lysis buffer containing 50 mM Tris (pH 7.5), 120 mM NaCl, 0.5% NP-40, 5 mM EDTA and protease and phosphatase inhibitors (Thermo Fisher Scientific). Total protein (500 μg) were incubated with 5 μg specific antibodies on shaking tables at 4 °C overnight, followed by incubation with 35 μl protein G-sepharose beads (Thermo Fisher Scientific) under the same experimental condition for 4 h. Pre-cooling PBS was employed to wash the immunoprecipitated samples for four times (5mins each). Next, proteins from the input and beads-antibody complex were examined by SDS-PAGE. GAPDH (Glyceraldehyde-3-phosphate dehydrogenase) was used as a control for input. Information for all antibodies is available in Additional file 1: Table S1.

### Statistical analysis

Data were expressed as mean ± SEM. Group differences were analyzed using independent Student’s *t*-test or analysis of variance (ANOVA). Log-rank (Mantel–Cox) test was adopted to determine the significance (*p* value) of patient overall survival status. All graphics and statistical analyses were performed using GraphPad Prism5 statistical software. Results were considered as statistically significant when *p* value was < 0.05.

## Results

### Expression of Gab1 is positively associated with BCa metastasis clinically

**Fig. 1:**
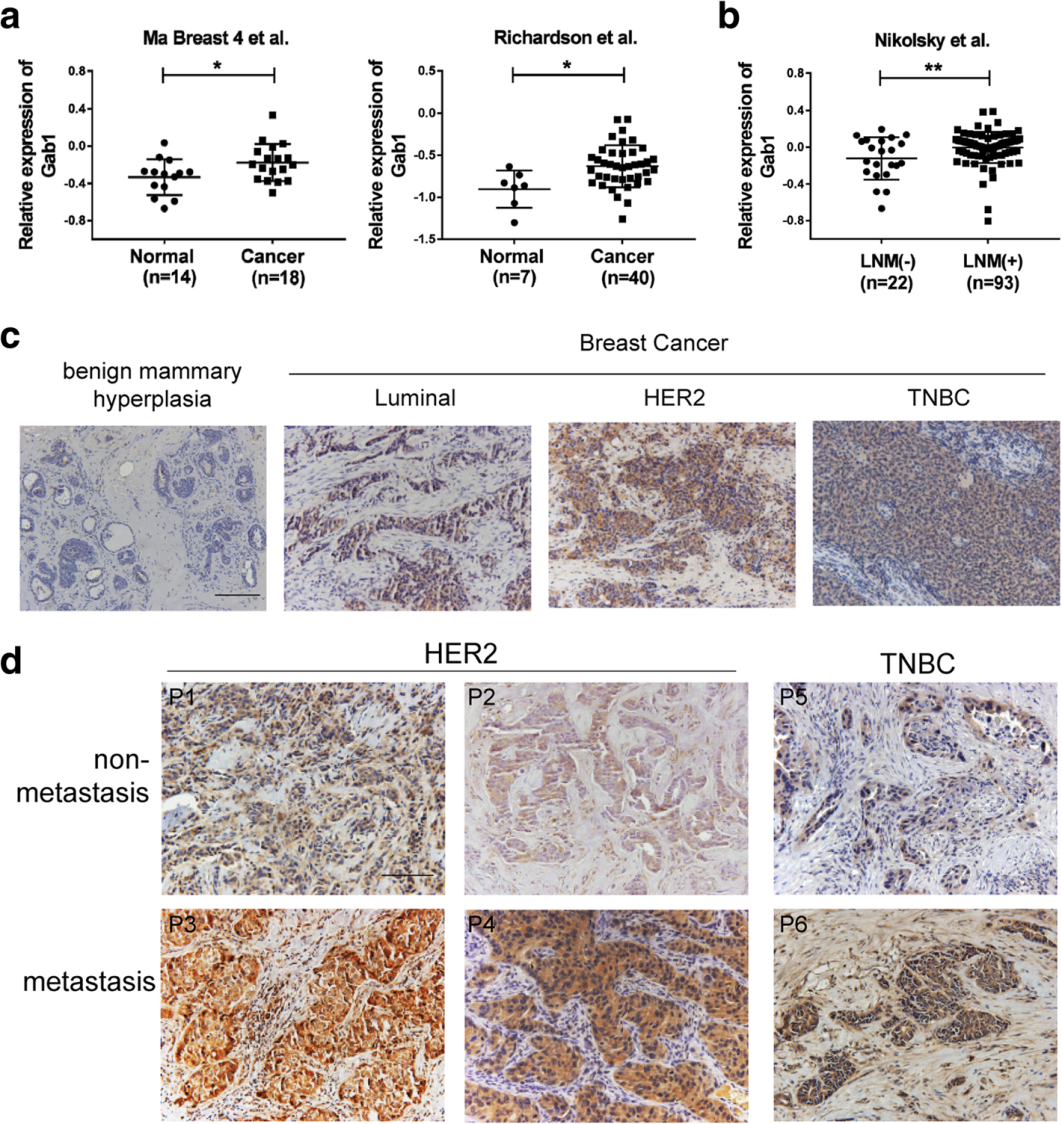
Expression of Gab1 is upregulated in metastatic BCa tissues. **a** Analysis of datasets from Oncomine database shows that Gab1 expression is upregulated in BCa tissues when compared to the normal mammary tissues. **b** Expression of Gab1 is significantly upregulated in BCa tissues with lymph node metastasis when compared to that with primary tumor only. **c** Expression of Gab1 is measured by IHC staining in tumor tissues from a luminal, HER2 or TNBC subtype BCa patient and in mammary tissue from a benign mammary hyperplastic control respectively. **d** Expression of Gab1 is measured by IHC staining in tumor tissues with or without metastasis from HER2 or TNBC subtype BCa patients. Scale Bar: 100 μm, P: patient, Data are presented as means ± SEM. *: *p* < 0.05, **: *p* < 0.01

To investigate expressional level of Gab1 in BCa, we first analyzed two independent datasets from Oncomine database (Ma Breast 4 et al. [16] and Richardson et al. [17]). Data from both of datasets indicated that expression of Gab1 was significantly elevated in BCa samples when compared to normal control samples (Fig. 1a). Next, we wondered whether expressional level of Gab1 is correlated with metastasis in BCa. By analyzing another Oncomine dataset (Nikolsky et al. [18]), we found that patients with lymph node metastasis (LNM) showed increased expression of Gab1, compared to patients without metastasis (Fig. 1b). To validate these results, we then examined Gab1 expression in patient samples collected from our hospital by western blot assay. We found that expression of Gab1 was indeed upregulated in BCa tissues (*n* = 8) when compared to the paired adjacent normal control tissues (Additional file 2: Figure S1a and Additional file 3: Table S1). Furthermore, we carried out IHC staining for Gab1 and IF co-staining for Gab1 and EpCAM to further determine Gab1 expression in these clinical tumor samples from three major subtypes of BCa, i.e. luminal BCa (*n* = 6 for IHC and n = 6 for IF), HER2 BCa (*n* = 6 for IHC and *n* = 6 for IF) and triple negative breast cancer (TNBC, *n* = 6 for IHC and *n* = 6 for IF), respectively (Additional file 2: Figure S1b, S1c and Additional file 3: Table S2, Table S3). Comparison to benign mammary hyperplastic control samples (*n* = 6 for IHC and *n* = 6 for IF), significantly elevated Gab1 expression was observed in all of the BCa subtypes (Fig. 1c, Additional file 2: Figure S1d). Importantly, in either HER2 BCa (*n* = 4) or TNBC subtype (*n* = 2) our IHC staining assessment confirmed a further upregulated Gab1 expression in metastatic samples (Fig. 1d and Additional file 3: Table S4). Support for our findings also came from the result of Oncomine data analysis (Ma Breast 3 et al. [19]), which showed a positive association of Gab1 expressional level with malignant grade progression in BCa (Additional file 2: Figure S1e). In addition, patients with high expressional level of Gab1 displayed a lower rate of overall survival via data assay using The Cancer Genome Atlas (TCGA) database (Additional file 2: Figure S1f). Taken together, these results indicate that expression of Gab1 is not only upregulated in BCa patients with malignant tumor growth and a poor prognosis but also positively associated with tumor metastasis.

### Elevated expression of Gab1 enhances BCa cell migration by dissociating the PAR complex in vitro

**Fig. 2:**
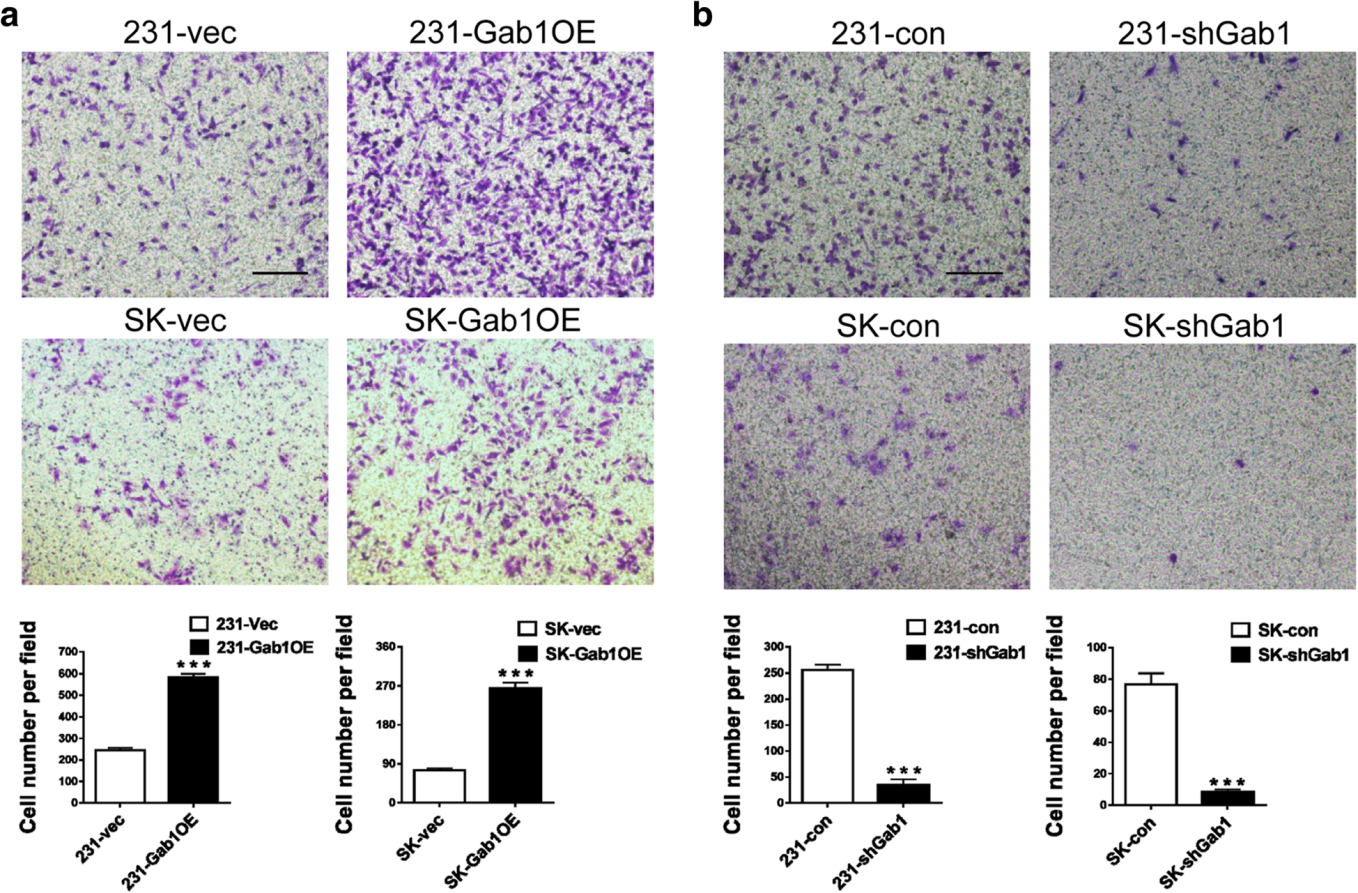
Gab1 overexpression promotes but Gab1 knockdown inhibits BCa cell migration in vitro. **a** Overexpression of Gab1 enhances cell migration of MDA-MB-231 and SK-BR3 cells by transwell assay. **b** Knockdown of Gab1 inhibits cell migration of MDA-MB-231 and SK-BR3 cells by transwell assay. Scale bars: 200 μm, Data are presented as means ± SEM. ***: *p* < 0.001

To investigate what role of Gab1 plays in regulation of BCa metastasis, we construct Gab1 stable overexpression and related control subclones in MDA-MB-231 (a TNBC cell line) and SK-BR3 (a HER2 BCa cell line) cells respectively (named 231-Gab1OE vs. 231-vec and SK-Gab1OE vs. SK-vec cell, see Additional file 2: Figure S2a). We also constructed Gab1 stable knockdown and related control subclones in the same cell lines (named 231-shGab1 vs. 231-con and SK-shGab1 vs. SK-con cell, see Additional file 2: Figure S2b). By transwell assay in vitro, we observed that overexpression of Gab1 increased cell migration to about 2.4 folds in 231-Gab1OE cells (582.3 ± 9.6 cells/field in 231-Gab1OE vs. 244.3 ± 6.386 cells/field in 231-vec, *p* < 0.001), and to about 3.5 folds in SK-Gab1OE cells (263.7 ± 8.2 cells/field in SK-Gab1OE vs 74.3 ± 3.2 cells/field in SK-vec, *p* < 0.001) when compared to the relevant controls respectively (Fig. 2a). In contrast, Gab1 knockdown decreased cell migration in 231-shGab1 cells (34.7 ± 6.1 cells/field in 231-shGab1 vs. 254 ± 6.6 cells/field in 231-con, *p* < 0.001) and SK-shGab1 cells (8.3 ± 0.9 cells/field in SK -shGab1 vs. 76.7 ± 4.1 cells/field in SK-con, *p* < 0.001) compared to relevant controls, respectively (Fig. 2b). In addition, overexpression or knockdown of Gab1 did not show a significant effect on cell proliferation in vitro (*p* = 0.168 upon Gab1 overexpression and *p* = 0.238 upon Gab1 knockdown, Additional file 2: Figure S2c and Additional file 4: Supplementary Methods). In order to exclude influence of Gab1 overexpression or knockdown on cell proliferation, we treated cells with Mitomycin C (10 μg/ml) and repeated our transwell assay. The result again confirmed that expression of Gab1 is positively associated with cell migration in vitro (Additional file 2: Figure S2d). Our wound healing assessment either under the treatment of Mitomycin C or not also revealed a similar result, that is, an improved migration upon stable overexpression of Gab1 but a repressed migration upon stable knockdown of Gab1 in both MDA-MB-231 and SK-BR3 cells respectively (Additional file 2: Figure S2e, S2f). Thus, these results indicate that upregulation of Gab1 expression enhances BCa cell migration in vitro.

**Fig. 3:**
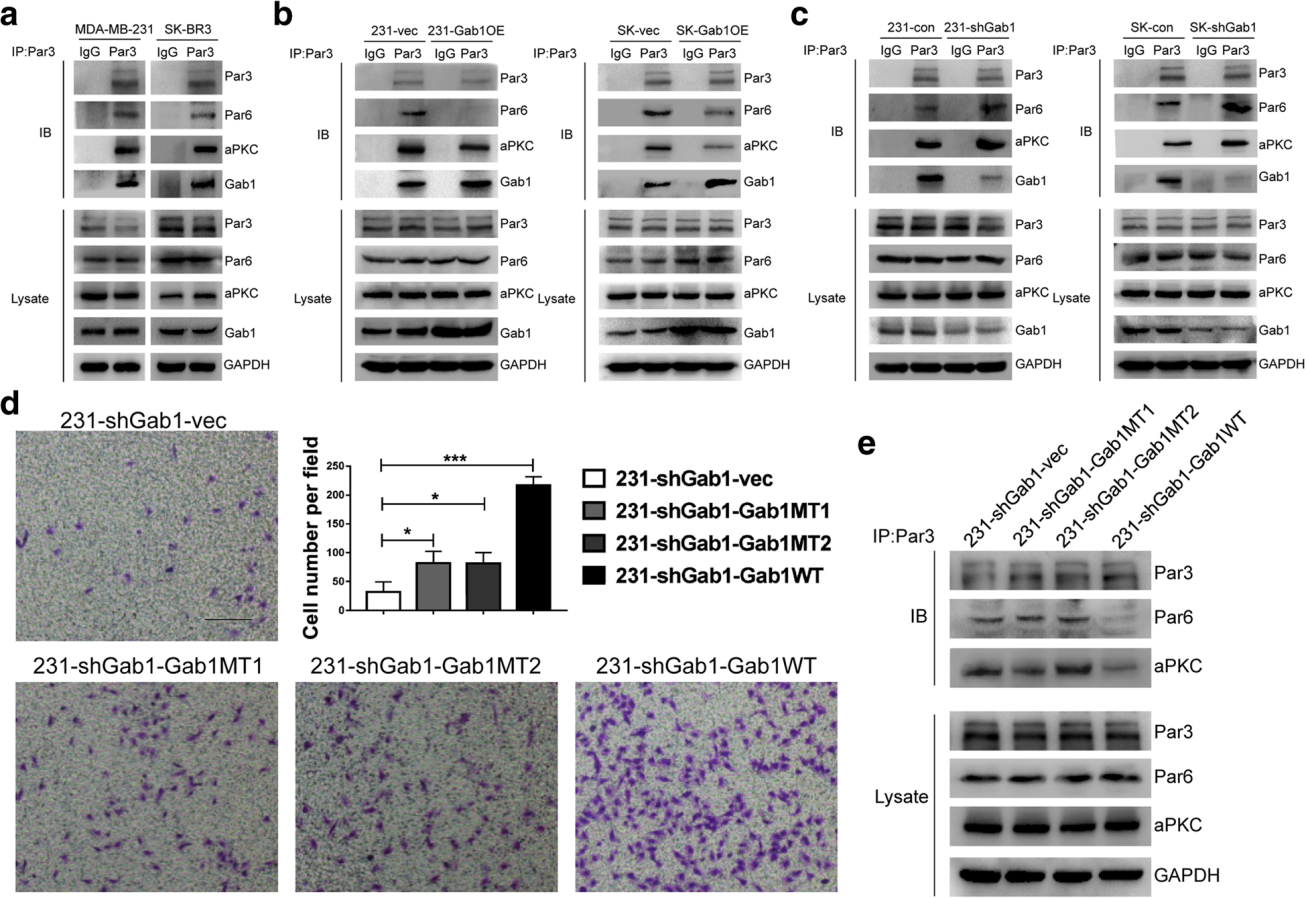
Gab1 overexpression dissociates the PAR complex. **a** Endogenous interaction of Par3 with Par6, aPKC and Gab1 is detected in MDA-MB-231 and SK-BR3 cells. **b** Overexpression of Gab1 inhibits the interaction of Par3 with Par6 and aPKC through an enhanced Gab1-Par3 interaction in both MDA-MB-231 and SK-BR3 cells. **c** Knockdown Gab1 interrupts the Gab1-Par3 interaction to fasten the interaction of Par3 with Par6 and aPKC in both MDA-MB-231 and SK-BR3 cells. **d** Re-expression of a fully functional but not a Par1b/Par3 binding domain deletion mutant Gab1 improves cell migration on the background of Gab1 knockdown. Scale bars: 200 μm **e** Re-expression of a fully functional but not a Par1b/Par3 binding domain deletion mutant Gab1 inhibits the interaction of Par3 with both Par6 and aPKC

In our previous work, we showed that dissociation of the PAR complex promotes prostate cancer metastasis [14] and that interaction of Gab1 with Par3 and Par1b can induce dissociation of the PAR complex in epithelial cells [15]. Considering these previous findings together with our results above, we herein wondered whether elevated expression of Gab1 enhances BCa cell migration via dissociating the PAR complex. By co-IP assay, interaction among components of PAR complex, i.e. Par3, Par6 and aPKC, as well as interaction of Gab1 with Par1b and Par3 were confirmed in both MDA-MB-231 and SK-BR3 cell lines (Fig. 3a and Additional file 2: Figure S3a). After overexpression of Gab1, we repeated co-IP assay and found that interaction of Gab1 with either Par1b or Par3 was enhanced but the interaction of Par3 with either Par6 or aPKC was impaired (Fig. 3b and Additional file 2: Figure S3b). Consistent with this observation, decreased co-staining of Par3 and Par6 by IF staining assay also indicated dissociation of the PAR complex (Additional file 2: Figure S3c, S3d). Reversely, knockdown of Gab1 repressed its interaction with both Par3 and Par1b and in turn restored interaction among Par3, Par6 and aPKC (Fig. 3c and Additional file 2: Figure S3e).

To clarify that upregulation of Gab1 dissociated the PAR complex via enhanced Gab1-Par3 and Gab1-Par1b interaction in BCa cells, we further constructed a series of subclone cell lines based on 231-shGab1 subclone cell line to re-expression of a Par1b binding domain deficient Gab1 mutant (named 231-shGab1-Gab1MT1), a Par3 binding domain deficient Gab1 mutant (named 231-shGab1-Gab1MT2), a synonymous mutation of Gab1 that antagonizes Gab1 shRNAs (named 231-shGab1-Gab1WT), or a vector control (named 231-shGab1-vec), respectively, by infecting a relevant mutant or functional Gab-1-carrying lentiviral vector as a restoration assay (Additional file 2: Figure S4a and S4b). By transwell assay, we found that cell migration was limitedly restored after re-expression of either Gab1MT1 or Gab1MT2 (81.7 ± 20.8 cells/field in 231-shGab1-MT1 vs. 31.6 ± 17.5 cells/field in 231-shGab1-vec, *p* < 0.05, 81.3 ± 19.0 cells/field in 231-shGab1-MT2 vs. 31.6 ± 17.5 cells/field in 231-shGab1-vec, *p* < 0.05). As comparison, re-expression of a fully functional Gab1 effectively restored cell migration (216.7 ± 15.3 cells/field in 231-shGab1-WT vs. 31.6 ± 17.5 cells/field in 231-shGab1-vec, *p* < 0.001), indicating that both Gab1-Par1b and Gab1-Par3 interaction were essential for the dissociation of the PAR complex (Fig. 3d and Additional file 2: Figure S4c). Furthermore, we repeated co-IP assay and found that re-expression of a fully functional Gab1 but not a Par1b binding or a Par3 binding deficient Gab1 significantly impaired interaction among the PAR complex components (Fig. 3e and Additional file 2: Figure S4d).

In addition, it has been recently shown that Gab1-Erk1/2 and Gab1/PI3K/Akt pathway act as main downstream of Gab1 to regulate cancer proliferation and metastasis in thyroid tumors and HCC [20, 21]. To determine whether these two pathways are involved in Gab1-upregulation mediated BCa metastasis, we herein performed expression assay of phosphorylation of both Erk1/2 and Akt. We found that phosphorylation of Erk1/2 remained unchanged regardless of Gab1 overexpression or knockdown. In comparison, we found that phosphorylation of Akt at Thr308 but not at Ser473 residue site was increased or decreased as a response to Gab1 overexpression or Gab1 knockdown in BCa cells, although it has been reported that both sites can be phosphorylated by the Gab1/PI3K/Akt pathway [22] (Additional file 2: Figure S5a and S5b). Notably, whereas both Par1b (MT1) and Par3 (MT2) binding deficient mutant Gab1s can restore phosphorylation of Akt on the background of Gab1 knockdown, both of them failed to restore BCa cell migration in vitro (Fig. 3d and Additional file 2: Figure S5c), indicating that activation of Akt is not essential for Gab1 upregulation-induced promotion of BCa cell migration. In addition, expression of p-Erk1/2 was similar no matter whether a mutant or a fully functional Gab1 was re-expressed as a rescue after Gab1 knockdown (Additional file 2: Figure S5c). Taken together, these findings indicate that upregulation of Gab1 dissociates the PAR complex, rather than activating either the Gab1-Erk1/2 or the Gab1/PI3K/Akt pathway, to enhance BCa cell migration as a mechanism.

### Dissociation of the PAR complex induces EMT in BCa

**Fig. 4:**
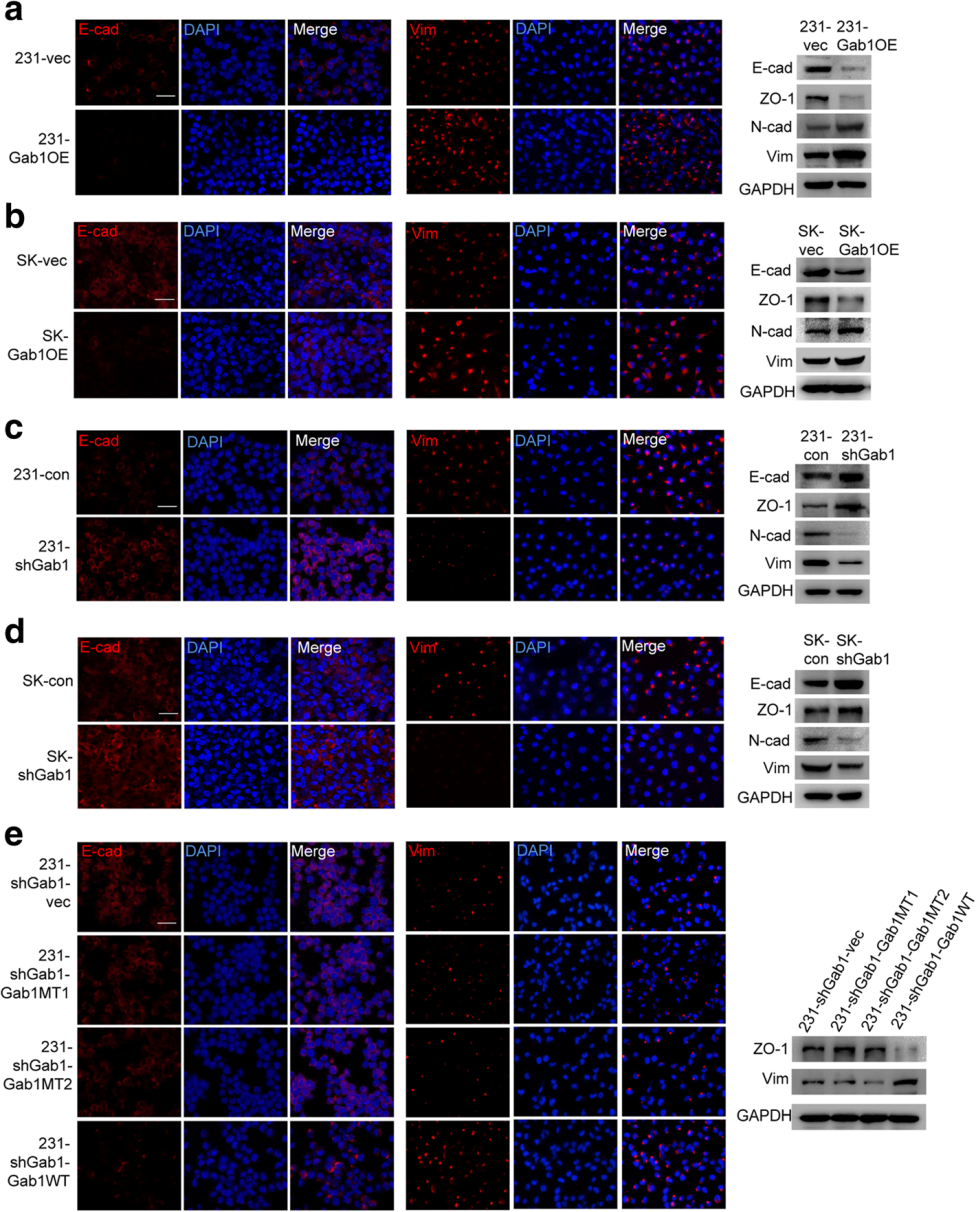
Gab1 overexpression promotes EMT in BCa cells. (**a**, **b**) Overexpression of Gab1 decreases the expression of E-cad and ZO-1 and increases the expression of N-cad and Vimentin in both MDA-MB-231 (**a**) and SK-BR3 (**b**) cells. (**c**, **d**) Knockdown of Gab1 increases the expression of E-cad and ZO-1 and decreases the expression of N-cad and Vimentin in both MDA-MB-231 (**c**) and SK-BR3 (**d**) cells. **e** Re-expression of a fully functional but not a Par1b/Par3 binding domain deletion mutant Gab1 restores the expression of Vimentin as well as represses the expression of E-cad and ZO-1 after Gab1 knockdown in MDA-MB-231 cells. Scale bars: 50 μm, Vim: Vimentin

Next, we wondered whether dissociation of the PAR complex can induce EMT in BCa, as what we observed in prostate cancer [14]. To this end, we measured protein expression level of well-known EMT makers such as E-cad, ZO-1, N-cad and Vimentin, by IF staining and western blot assay. We found that expression of E-cad and ZO-1 was significantly decreased but expression of N-cad and Vimentin was significantly increased upon Gab1 overexpression in both MDA-MB-231 and SK-BR3 cell lines, indicating an induction of EMT (Fig. 4a, b and Additional file 2: Figure S6a). As expected, stable knockdown of Gab1 had an effect on repression of EMT, which was evidenced by an epithelial-like morphological change and an elevation of E-cad and ZO-1 expression along with a repression of N-cad and Vimentin expression (Fig. 4c, d and Additional file 2: Figure S6b). Moreover, restoration of EMT was observed when re-expression of a fully functional Gab1 on the background of Gab1 knockdown (Fig. 4e and f). Therefore, our data suggest that dissociation of the PAR complex can also induce EMT in BCa.

### Upregulation of Gab1 promotes BCa metastasis in vivo

**Fig. 5:**
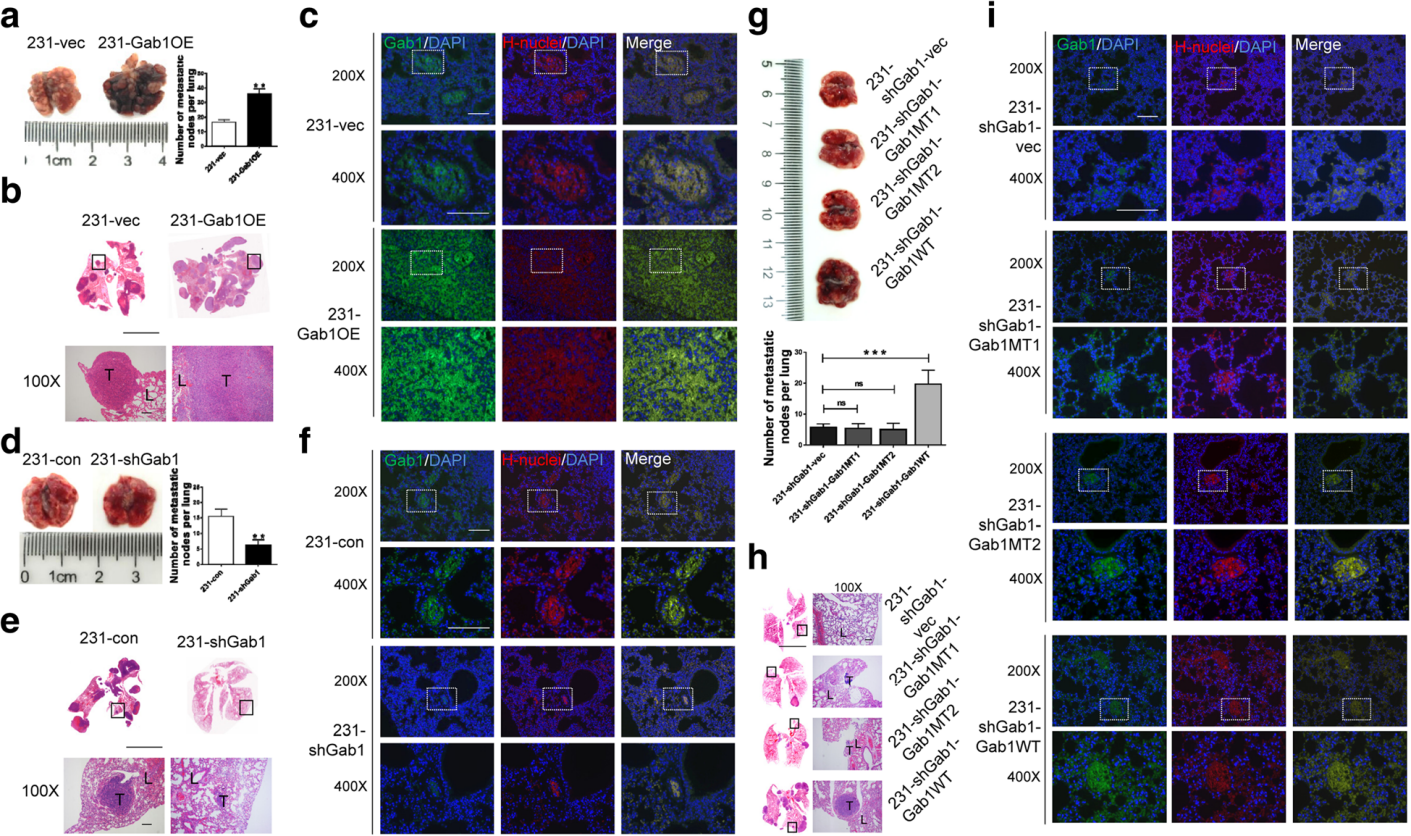
Upregulated Gab1 enhances BCa metastasis in vivo*.* **a** Overexpression of Gab1 increases the number of metastatic nodes in lung tissues. **b** Histopathological assessment in lung tissues from 231-vec or 231-Gab1OE cells inoculated mice by H&E staining. **c** Gab1 expression is detected in lung metastatic nodes by co-staining of Gab1 and H-nuclei. **d** Knockdown of Gab1 decreases the number of metastatic nodes in lung tissues. **e** Histopathological assessment in lung tissues from 231-con or 231-shGab1 cells inoculated mice by H&E staining. **f** Downregulated Gab1 expression was identified in lung tissues from 231-shGab1 cells inoculated mice. **g** Re-expression of a fully functional but not a Par1b/Par3 binding domain deletion mutant Gab1 improves lung metastasis of BCa. **h** Histopathological assessment in lung tissues from 231-shGab1-vec, 231-shGab1-Gab1MT1, 231-shGab1-Gab1MT2 or 231-shGab1-Gab1WT cells inoculated mice by H&E staining. **i** Gab1 expression is increased after re-expression of a fully functional but not a Par1b/Par3 binding domain deletion mutant Gab1 upon Gab1 knockdown. Scale Bar: 1 cm for whole tissue scanning and 100 μm for micrograph in (**b**), (**e**) and (**h**), Scale Bar: 100 μm for IF staining in (**c**), (**f**) and (**i**), H-nuclei: human nuclei, L: lung, T: tumor,**: *p* < 0.01

To validate our hypothesis that upregulation of Gab1 promotes BCa metastasis in vivo, we developed related mouse models in female nude mice by intraveneous injection of relevant BCa subclone cell lines through the tail vein. Four weeks later, lung metastasis of BCa were observed in mice in the group of inoculation of either 231-vec (*n* = 5) or 231-Gab1OE cells (*n* = 5). Consistent with our findings in vitro, Gab1 overexpression significantly increased number of metastatic nodes in lung tissues (36.7 ± 2.8 nodes in 231-Gab1OE group vs. 17.5 ± 1.6 nodes in 231-vec group, *p* < 0.01, see Fig. 5a, b and Additional file 2: Figure S7a, S7b). By IF staining assay, upregulation of Gab1 was detected in lung tissues from 231-Gab1OE cell-inoculated mice (Fig. 5c and Additional file 2: Figure S7c). As expected, Gab1 knockdown significantly repressed BCa lung metastasis, as evidenced by a decreased number of metastatic nodes (6.7 ± 1.8 nodes in 231-shGab1, *n* = 5, vs. 15.5 ± 2.2 nodes in 231-con group, *n* = 5, *p* < 0.01, see Fig. 5d, e and Additional file 2: Figure S7d, S7e) along with an attenuated Gab1 staining in lung tissues (Fig. 5f). Furthermore, restoration of a fully functional but not a Par1b binding deficient or a Par3 binding deficient Gab1 reversed the metastasis repressive phenotype induced by Gab1 knockdown and represented an enhanced lung metastasis (Fig. 5g to i). In addition, we developed subcutaneous xenograft models by inoculation of relevant cells into the flank of mouse leg to investigate whether overexpression (*n* = 5) or knockdown of Gab1 (*n* = 5) can alter cell viability (Additional file 4: Supplementary Methods). Similar to the result of cell proliferation assay in vitro, overexpression or knockdown of Gab1 did not show a significant effect on tumor survival (*p* = 0.167 upon Gab1 overexpression and *p* = 0.382 upon Gab1 knockdown), indicating that upregulation of Gab1 promotes metastasis by enhancing migratory behaviors of BCa but not by increasing cell viability (Additional file 2: Figure S7f). For further confirmation, we also developed orthotopic xenograft models by inoculation of relevant cells into the fat pad of the mouse breast (Additional file 4: Supplementary Methods). Ten weeks after inoculation, whereas macroscopically visible metastases in the lung were not observed due to a limited monitoring time, we found that the orthotopic tumor growth was similar between 231-vec and 231-Gab1OE group but the micro-invasion into adjacent normal tissues was enhanced in 231-Gab1OE group, as evidenced by a tissue H&E staining and by a reduced laminin IHC staining in tissues collected from 231-Gab1OE injected mice, respectively (Additional file 2: Figure S7 g-S7i). Collectively, these data together indicate that elevated expression of Gab1 promotes BCa metastasis in vivo.

## Discussion

Metastasis is a major challenge in BCa treatment. An early diagnosis and intervention for metastatic BCa is currently unavailable due to limited understanding of the underlying molecular mechanism [23, 24]. In this study, we for the first time provide experimental evidence that not only elevated expression of Gab1 is positively correlated with BCa, but also its expression is further upregulated in tissues from patients with metastatic BCa. Through gain-of-function and loss-of-function assays, we demonstrated that Gab1 overexpression promotes BCa metastasis via a new mechanism, that is, dissociation of the PAR complex to induce EMT. Based on these findings, we propose a potential diagnostic and therapeutic strategy for BCa metastasis by targeting Gab1.

Up to date, although several genes have been implicated as subtype-specific diagnostic markers and therapeutic targets for BCa metastasis (e.g., HMGA1 and CDCA7 for TNBC metastasis [25, 26]), universal target genes for diagnosis and treatment of tumor metastasis in multiple subtypes of BCa are still required. In current study, by combining the analysis of public available datasets with our own experimental results, we demonstrated that Gab1 is not only overexpressed in primary BCa clinical samples but also further elevated in metastatic BCa of both HER2 and TNBC subtypes. Furthermore, through Gab1 knockdown and consequential restoration assays in vitro and in vivo in HER2 BCa (SK-BR3) cells and TNBC (MDA-MB-231) cells, we also demonstrated that repression of Gab1 expression is an effective approach to inhibit BCa metastasis, implicating a promising therapeutic strategy against metastatic BCa of both subtypes by targeting Gab1. In agreement with our findings, repression of Gab1 expression has been previously shown to inhibit metastasis in multiple cancers including colorectal cancer, hepatocellular carcinoma, ovarian cancer and hilar cholangiocarcinoma [21, 27–29]. In addition, a recent bioinformatic study indicated that a structure-based approach to target the pleckstrin homology (PH) domain of Gab1 represents a potential tumor-specific cytotoxicity against MDA-MB-231 and T47D BCa cell lines in vitro [30]. Therefore, combined with these previous reports, our findings reinforce the notion that Gab1 may serve as a universal target gene not only for diagnosis but also for therapy of BCa metastasis regardless of its subtype.

Mechanistically, we for the first time demonstrated that elevated expression of Gab1 dissociates the PAR complex to induce EMT to promote BCa metastasis. In particular, we found that overexpression of Gab1 enhances Gab1-Par1b interaction as well as Gab1-Par3 interaction, causing an attenuated Par3-Par6 interaction and Par3-aPKC interaction and in turn leading to a dissociation of the PAR complex. For further confirmation of our hypothesis, we re-expressed either a Par1b/Par3 binding domain deletion mutant Gab1, or a fully functional Gab1 for a restoration assay on the background of Gab1 knockdown. As expected, re-expression of a Gab1 with either Par1b or Par3 binding domain deletion fails to reverse the phenotype of repressive metastasis and keeps a limited interaction with Par1b or Par3, both of which are caused by Gab1 knockdown. In contrast, re-expression of a fully functional Gab1 effectively improves BCa cell migration in vitro and BCa metastasis in vivo by restoring Gab1-Par1b interaction and Gab1-Par3 interaction, which verifies that dissociation of the PAR complex caused by Gab1 overexpression can promote BCa metastasis. Moreover, while Par3 deficiency is previously shown to promote lung metastasis in BCa via inactivation of the JAK/Stat3 signaling pathway to increase production of MMP9 [31], we demonstrated in current study that enhanced Gab1-Par3 interaction does not change Par3 expression but dissociates the PAR complex to induce EMT as a novel mechanism to promote BCa metastasis.

Our current study argues against a possibility that canonical Gab1/PI3K/Akt and/or Gab1/Erk pathway is a parallel mechanism for the regulation of BCa metastasis. Although a recent study revealed that activated Gab1 facilitates the activation of its downstream Shp2-Erk pathway to promote ovarian cancer metastasis [32], the present experiments showed that no significant change of phosphorylation of Erk is observed no matter whether Gab1 is overexpressed or knocked down, indicating that overexpression of Gab1 promotes BCa metastasis in an Erk activity-independent manner. In addition, it has been previously reported that Gab1 regulates tumor cell growth, apoptosis and invasion through the VEGFR-2/Gab1/PI3K/Akt signaling in hilar cholangiocarcinoma cells [29]. In this study, an increased or a decreased phosphorylation of Akt at Thr308 site can be observed after Gab1 overexpression or knockdown. However, results from our restoration assay revealed that although phosphorylation of Akt is increased by re-expression of either a Par1b/Par3 binding domain deletion mutant Gab1 or a fully functional Gab1, a repressive phenotype of metastasis after Gab1 knockdown is reversed only when a fully functional Gab1 was re-expressed. Taken together, these findings strongly support the notion that Gab1 exerts its function on promotion of BCa metastasis independent upon activation of Akt and Erk. Further investigation, such as employing a spontaneous mammary carcinogenesis and tumor metastasis transgenic mouse model for phenotype observation and mechanism exploration, is still required to be carried out for potential application of Gab1 in diagnosis and therapy of metastatic BCa.

## Conclusion

Taken together, our study underscores the biological and clinical significance of Gab1 in BCa. Elevated expression of Gab1 promotes BCa metastasis via dissociating the PAR complex to induce EMT. Knockdown of Gab1 attenuates its interaction with Par1b or Par3, leading to a stabilization of the PAR complex to inhibit metastasis. Measurement of Gab1 expression might provide a prediction of BCa metastasis. Pharmaceutical intervention to repress Gab1 expression or in combination with other therapeutic approaches might provide a more promising strategy to inhibit BCa metastasis.

## Additional files

Additional file 1: **Table S1.** Antibodies used in this study. (PDF 77 kb)

Additional file 2: **Figure S1.** Expression of Gab1 is upregulated in BCa tissues. (a) Protein expression of Gab1 is increased in 6 of 8 BCa tissues when compared to the adjacent normal tissues (N: normal tissues, T: tumor tissues). (b) Gab1 antibody used for IHC staining is checked by no-primary antibody control assay. Scale Bar: 100 μm (c) Primary antibodies of Gab1 and EpCAM used for IF staining are checked by no-primary antibody control assay. Scale Bar: 50 μm (d) Expression of Gab1 is upregulated in BCa tissues by IF co-staining for Gab1 and EpCAM. Scale Bar: 50 μm (e) Expression of Gab1 is positively associated with tumor progression by data analysis from Oncomine database. (f) Elevated expression of Gab1 predicts a poor survival by data analysis from TCGA database. Data are presented as means ± SEM. Overall survival was analyzed by log-rank test.*: *p* < 0.05, ***: *p* < 0.001 **Figure S2.** Overexpression of Gab1 promotes but knockdown of Gab1 inhibits BCa cell migration in vitro by wound healing assay. (a) A fully functional Gab1-carrying lentivirus is infected for stable overexpression of Gab1 in both MDA-MB-231 and SK-BR3 cells. (b) Lentivirus that carries independent Gab1 shRNA#1~#3 respectively is infected for knockdown of Gab1 in both MDA-MB-231 and SK-BR3 cells. Gab1 shRNA#2 exerts an optimal inhibitory effect on Gab1 expression and thus is used for all of the related experiments in this study. (c) Overexpression or knockdown of Gab1 does not show a significant influence on cell proliferation in vitro. (d) Gab1 overexpression enhances but Gab1 knockdown inhibits BCa cell migration in vitro after treatment with Mitomycin C (10 μg/ml) for 1 h by transwell assay. Scale bars: 200 μm (e, f) Gab1 overexpression enhances but Gab1 knockdown inhibits BCa cell migration in vitro under the condition of either Mitomycin C (10 μg/ml) treatment (f) or not (e) by wound healing assay. Scale bars: 100 μm, Data are presented as means ± SEM. ***: *p* < 0.001 **Figure S3.** Overexpression of Gab1 enhances while knockdown of Gab1 attenuates the interaction between Gab1 and Par1b. (a) Endogenous interaction of Gab1 with Par1b and Par3 is confirmed by co-IP assay in both MDA-MB-231 and SK-BR3. (b) Stable overexpression of Gab1 enhances its interaction with Par1b in both MDA-MB-231 and SK-BR3. (c) Primary antibodies recognizing Par3 and Par6 used for IF staining are checked by no-primary antibody control assay. Scale Bar: 50 μm (d) IF staining assay shows that the interaction of Par3 and Par6 is attenuated upon Gab1 overexpression. White arrow: typical cells with or without co-staining of Par3 and Par6 in the control or Gab1 overexpressed cells, Scale bars: 50 μm (e) Knockdown of Gab1 attenuates its interaction with Par1b in both MDA-MB-231 and SK-BR3. **Figure S4.** Deletion of Par1b or Par3 binding domain in Gab1 inhibits the interaction of Gab1 with Par1b or Par3. (a) Structure schematic of Par1b or Par3 binding domain deletion mutant Gab1. (b) Expression of wild type or mutant Gab1 is detected by western blot assay. (c) Cell migration is restored by re-expression of a fully functional but not a mutant Gab1 after treatment with Mitomycin C (10 μg/ml) for 1 h. (d) Par1b or Par3 binding domain deficiency inhibits the interaction of Gab1 with Par1b or Par3 on the background of Gab1 knockdown. Scale bars: 200 μm, Data are presented as means ± SEM. **: *p* < 0.01, ***: *p* < 0.001 **Figure S5.** Expression of phosphorylated Akt and Erk after Gab1 overexpression or knockdown. (a) Gab1 overexpression enhances phosphorylation of Akt at Thr308 but not at Ser473 site, while it causes no influence on the phosphorylation of Erk1/2 in MDA-MB-231 cells. (b) Knockdown of Gab1 inhibits phosphorylation of Akt at Thr308 but not at Ser473 site, while it causes no influence on the phosphorylation of Erk1/2 in MDA-MB-231 cells. (c) Re-expression of a fully functional Gab1 but not a Par1b/Par3 binding domain deletion mutant Gab1 activates the phosphorylation of Akt, while either fully functional or mutant Gab1 has no effect on the phosphorylation of Erk1/2. **Figure S6.** Overexpression of Gab1 induces EMT in BCa. (a) Primary antibodies against E-cad and Vimentin are checked by no-primary antibody control assay. Scale Bar: 50 μm (b) Overexpression of Gab1 induces a mesenchymal-like morphological change and knockdown of Gab1 induces an epithelial-like morphological change in BCa cells. Scale Bar: 100 μm for lower magnification; 50 μm for higher magnification (up-right) **Figure S7.** Upregulation of Gab1 promotes BCa metastasis in vivo. (a) Overexpression of Gab1 increases the number of metastatic nodes in lung. (b) Histopathological assessment in lung tissues from 231-vec or 231-Gab1OE group by H&E staining. (c) Primary antibodies against Gab1 and H-nuclei are checked by no-primary antibody control assay in mouse lung tissues. (d) Knockdown of Gab1 decreases the number of metastatic nodes in lung. (e) Histopathological assessment in lung tissues from 231-con or 231-shGab1 group by H&E staining. (f) Overexpression or knockdown of Gab1 does not display a significant influence on tumor growth in vivo by subcutaneous xenograft models. (g) The orthotopic tumor growth is similar between 231-vec and 231-Gab1OE group by orthotopic xenograft models. (h) Primary antibody against laminin is checked by no-primary antibody control assay in mouse lung tissues. (i) The micro-invasion into adjacent normal tissues is enhanced in 231-Gab1OE group. Scale Bar: 1 cm for whole tissue scanning and 100 μm for micrograph in (b) and (e), Scale Bar: 100 μm for IF staining in (c) and for IHC staining in (h) and (i), H-nuclei: human nuclei, L: lung, T: tumor. (PDF 4076 kb)

Additional file 3: **Table S1.** Clinical data from patients for western blot assay (Fig. S1a). **Table S2.** Clinical data from BCa patients and benign mammary hyperplastic controls for IHC staining (Fig. 1c). **Table S3.** Clinical data from BCa patients and benign mammary hyperplastic controls for IF staining (Figure S1d). **Table S4.** Clinical data from patients with metastatic or primary BCa for IHC staining (Fig. 1d). (PDF 77 kb)

Additional file 4: Supplementary methods. (PDF 70 kb)

## Abbreviations

aPKC: Atypical protein kinase C
BCa: Breast cancer
CDCA7: Cell division cycle associated 7
co-IP: Co-immunoprecipitation
EMT: Epithelial-to-mesenchymal transition
FGFR: Fibroblast growth factor receptor
Gab: Grb2-Associated binding protein
GAPDH: Glyceraldehyde-3-phosphate dehydrogenase
HCC: Hepatocellular carcinoma
HMGA1: High mobility group AT-hook 1
IF: Immunofluorescence
IHC: Immunohistochemical
LNM: Lymph node metastasis
MMP: Matrix metalloproteinase
PAR complex: Partitioning defective complex
PH domain: Pleckstrin homology domain
PI3K: Phosphatidylinositol-4,5-bisphosphate 3-kinase
TNBC: Triple negative breast cancer
VEGFR: Vascular endothelial growth factor receptor
