# Supplementary material for: Elevated expression of Gab1 promotes breast cancer metastasis by dissociating the PAR complex
Source: J Exp Clin Cancer Res. 2019 Jan 21;38:27. doi: 10.1186/s13046-019-1025-2 (PMC6341703; doi:10.1186/s13046-019-1025-2)
Supplement: Supplementary file 1 — Table S1. Antibodies used in this study. (PDF 77 kb) [file 13046_2019_1025_MOESM1_ESM.pdf]

Additional file 1:

Table S1: Antibodies used in this study.

Antibodies	Company	Catalog	Application			
			**WB**	**IF**	**IHC**	**co-IP**
Akt	Cell Signaling Technology	#4691	√			
aPKC	Santa Cruz	sc-216	√			
E-cad	Abcam	ab128804	√	√		
EpCAM	Cell Signaling Technology	#2929	√	√	√	
Erk1/2	Cell Signaling Technology	#4695	√			
Gab1	Abcam	ab59362	√	√	√	
Gab1	Millipore	06-579				√
GAPDH	Cell Signaling Technology	#2118	√			
H-nuclei	Millipore	MAB1281		√		
Laminin	Abcam	ab11575			√	
N-cad	Cell Signaling Technology	#13116	√			
Par1b	Cell Signaling Technology	#9118	√			
Par3	Millipore	07-330	√	√		√
Par6	Life Products&Services	AB60695a	√	√		
Phospho-Akt(Thr308)	Abcam	ab38449	√			
Phospho-Akt(Ser473)	Cell Signaling Technology	#4060	√			
Phospho-Erk1/2	Cell Signaling Technology	#9101	√			
Vimentin	Cell Signaling Technology	#5741	√	√		
ZO-1	Thermo Fisher Scientific	33-9100	√			
