# Supplementary material for: Elevated expression of Gab1 promotes breast cancer metastasis by dissociating the PAR complex
Source: J Exp Clin Cancer Res. 2019 Jan 21;38:27. doi: 10.1186/s13046-019-1025-2 (PMC6341703; doi:10.1186/s13046-019-1025-2)
Supplement: Supplementary file 2 — Figure S1. Expression of Gab1 is upregulated in BCa tissues. (a) Protein expression of Gab1 is increased in 6 of 8 BCa tissues when compared to the adjacent normal tissues (N: normal tissues, T: tumor tissues). (b) Gab1 antibody used for IHC staining is checked by no-primary antibody control assay. Scale Bar: 100 μm (c) Primary antibodies of Gab1 and EpCAM used for IF staining are checked by no-primary antibody control assay. Scale Bar: 50 μm (d) Expression of Gab1 is upregulated in BCa tissues by IF co-staining for Gab1 and EpCAM. Scale Bar: 50 μm (e) Expression of Gab1 is positively associated with tumor progression by data analysis from Oncomine database. (f) Elevated expression of Gab1 predicts a poor survival by data analysis from TCGA database. Data are presented as means ± SEM. Overall survival was analyzed by log-rank test.*: p < 0.05, ***: p < 0.001 Figure S2. Overexpression of Gab1 promotes but knockdown of Gab1 inhibits BCa cell migration in vitro by wound healing assay. (a) A fully functional Gab1-carrying lentivirus is infected for stable overexpression of Gab1 in both MDA-MB-231 and SK-BR3 cells. (b) Lentivirus that carries independent Gab1 shRNA#1~#3 respectively is infected for knockdown of Gab1 in both MDA-MB-231 and SK-BR3 cells. Gab1 shRNA#2 exerts an optimal inhibitory effect on Gab1 expression and thus is used for all of the related experiments in this study. (c) Overexpression or knockdown of Gab1 does not show a significant influence on cell proliferation in vitro. (d) Gab1 overexpression enhances but Gab1 knockdown inhibits BCa cell migration in vitro after treatment with Mitomycin C (10 μg/ml) for 1 h by transwell assay. Scale bars: 200 μm (e, f) Gab1 overexpression enhances but Gab1 knockdown inhibits BCa cell migration in vitro under the condition of either Mitomycin C (10 μg/ml) treatment (f) or not (e) by wound healing assay. Scale bars: 100 μm, Data are presented as means ± SEM. ***: p < 0.001 Figure S3. Overexpression of Gab1 enhances [file 13046_2019_1025_MOESM2_ESM.pdf]

Additional file 2:

Figure S1

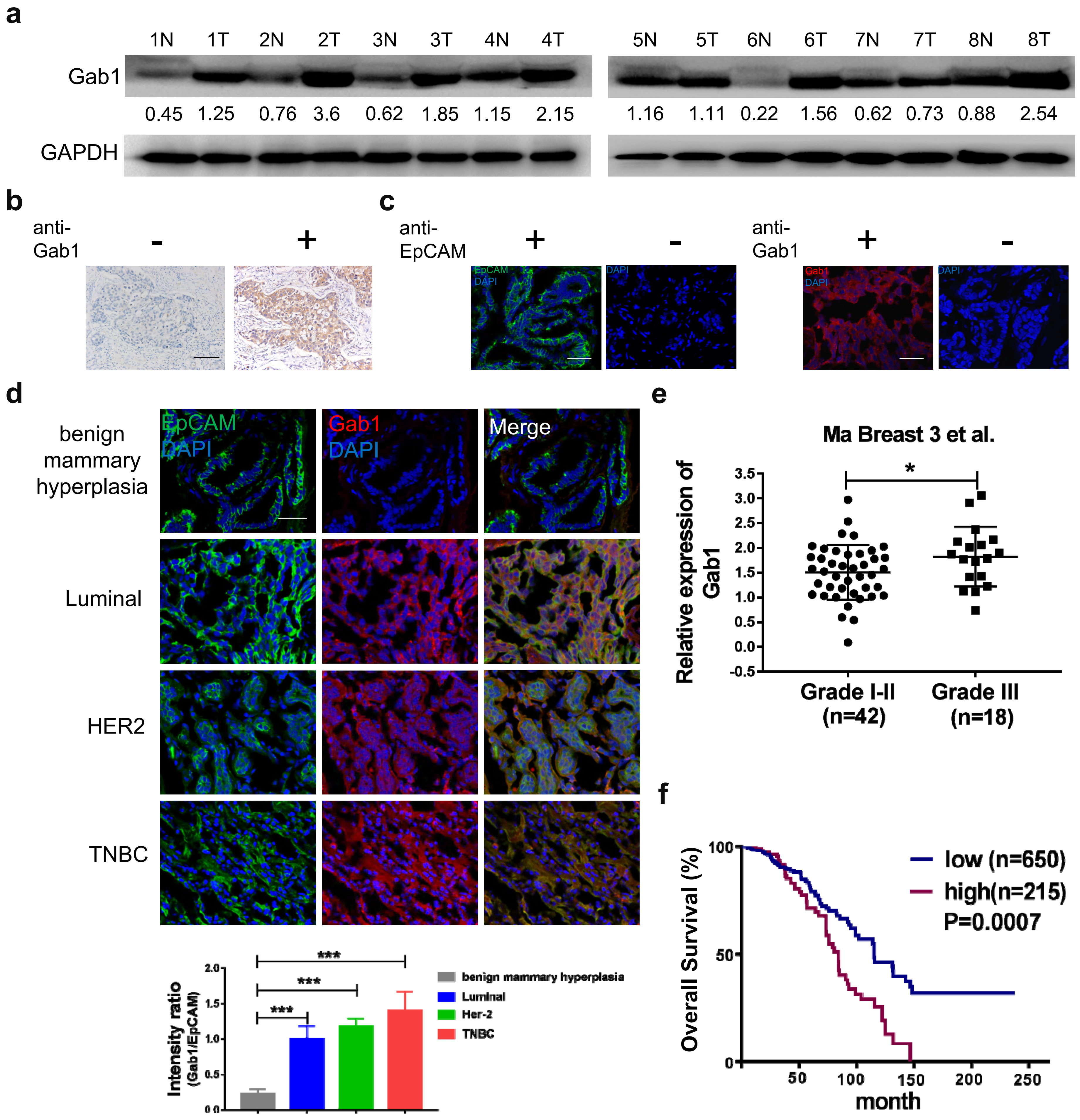

Figure S1. Expression of Gab1 is upregulated in BCa tissues. (a) Protein expression of Gab1 is increased in 6 of 8 BCa tissues when compared to the adjacent normal tissues (N: normal tissues, T: tumor tissues). (b) Gab1 antibody used for IHC staining is checked by no-primary antibody control assay. Scale Bar: 100 μ m (c) Primary antibodies of Gab1 and EpCAM used for IF staining are checked by no-primary antibody control assay. Scale Bar: 50 μ m (d) Expression of Gab1 is upregulated in BCa tissues by IF co-staining for Gab1 and EpCAM. Scale Bar: 50 μ m (e) Expression of Gab1 is positively associated with tumor grade progression by data analysis from Oncomine database. (f) Elevated expression of Gab1 predicts a poor survival by data analysis from TCGA database. Data are presented as means ± SEM. Overall survival was analyzed by log-rank test. ⁎: $p < 0.05$, ⁎⁎⁎: $p < 0.001$

Figure S2

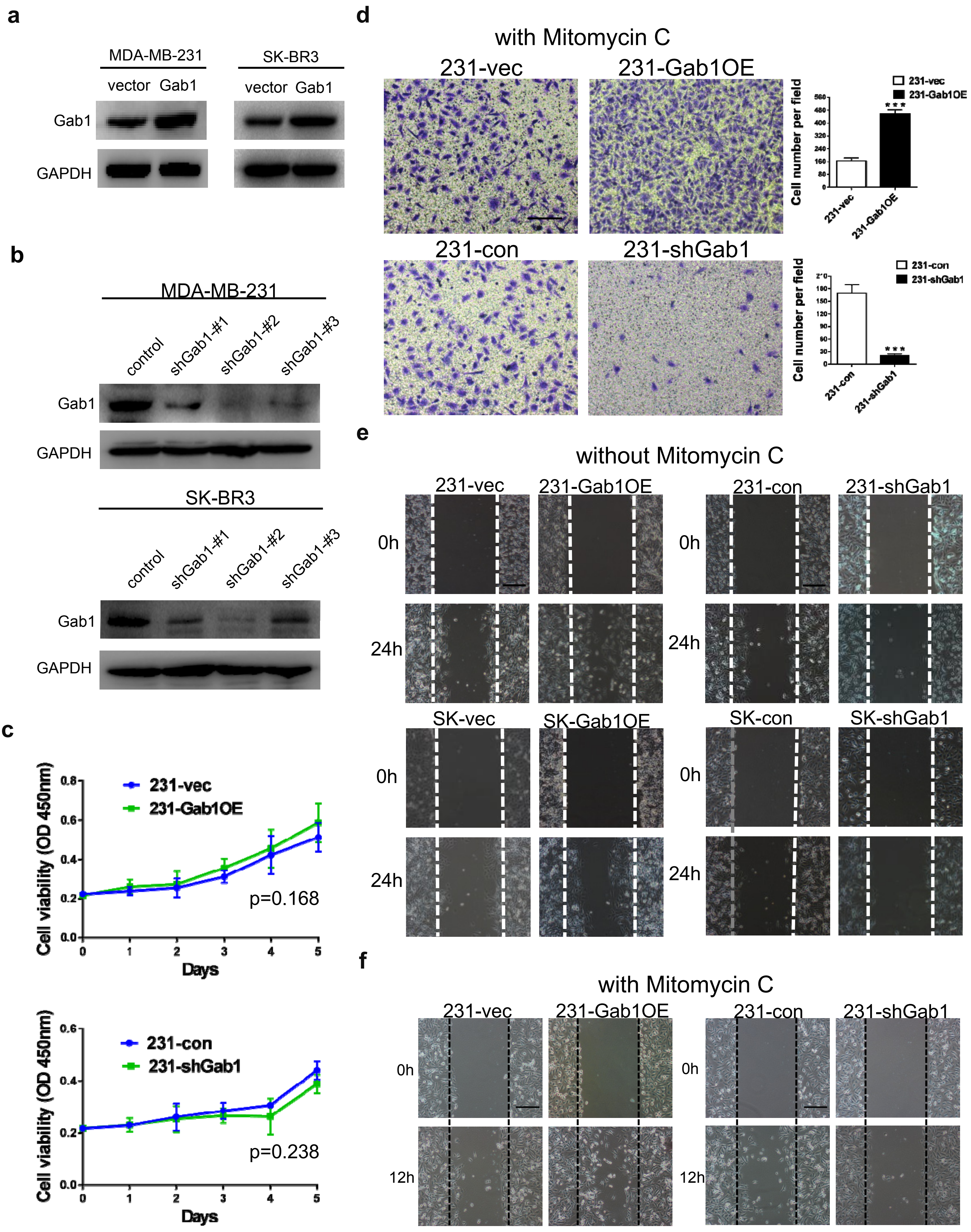

a
MDA-MB-231
SK-BR3
vector Gab1
vector Gab1
Gab1
GAPDH
b
MDA-MB-231
control
shGab1-#1
shGab1-#2
shGab1-#3
Gab1
GAPDH
SK-BR3
control
shGab1-#1
shGab1-#2
shGab1-#3
Gab1
GAPDH
c
231-vec
231-Gab1OE
Cell viability (OD 450nm)
Days
p=0.168
231-con
231-shGab1
Cell viability (OD 450nm)
Days
p=0.238
d
with Mitomycin C
231-vec
231-Gab1OE
231-con
231-shGab1
Cell number per field
e
without Mitomycin C
231-vec
231-Gab1OE
231-con
231-shGab1
0h
24h
SK-vec
SK-Gab1OE
SK-con
SK-shGab1
0h
24h
f
with Mitomycin C
231-vec
231-Gab1OE
231-con
231-shGab1
0h
12h

Figure S2. Overexpression of Gab1 promotes but knockdown of Gab1 inhibits BCa cell migration *in vitro* by wound healing assay. (a) A fully functional Gab1-carrying lentivirus is infected for stable overexpression of Gab1 in both MDA-MB-231 and SK-BR3 cells. (b) Lentivirus that carries independent Gab1 shRNA#1~#3 respectively is infected for knockdown of Gab1 in both MDA-MB-231 and SK-BR3 cells. Gab1 shRNA#2 exerts an optimal inhibitory effect on Gab1 expression and thus is used for all of the related experiments in this study. (c) Overexpression or knockdown of Gab1 does not show a significant influence on cell proliferation *in vitro*. (d) Gab1 overexpression enhances but Gab1 knockdown inhibits BCa cell migration *in vitro* after treatment with Mitomycin C (10μg/ml) for 1 h by transwell assay. Scale bars: 200μm (e, f) Gab1 overexpression enhances but Gab1 knockdown inhibits BCa cell migration *in vitro* under the condition of either Mitomycin C (10μg/ml) treatment (f) or not (e) by wound healing assay. Scale bars: 100μm, Data are presented as means ± SEM. ＊＊＊: $p < 0.001$

Figure S3

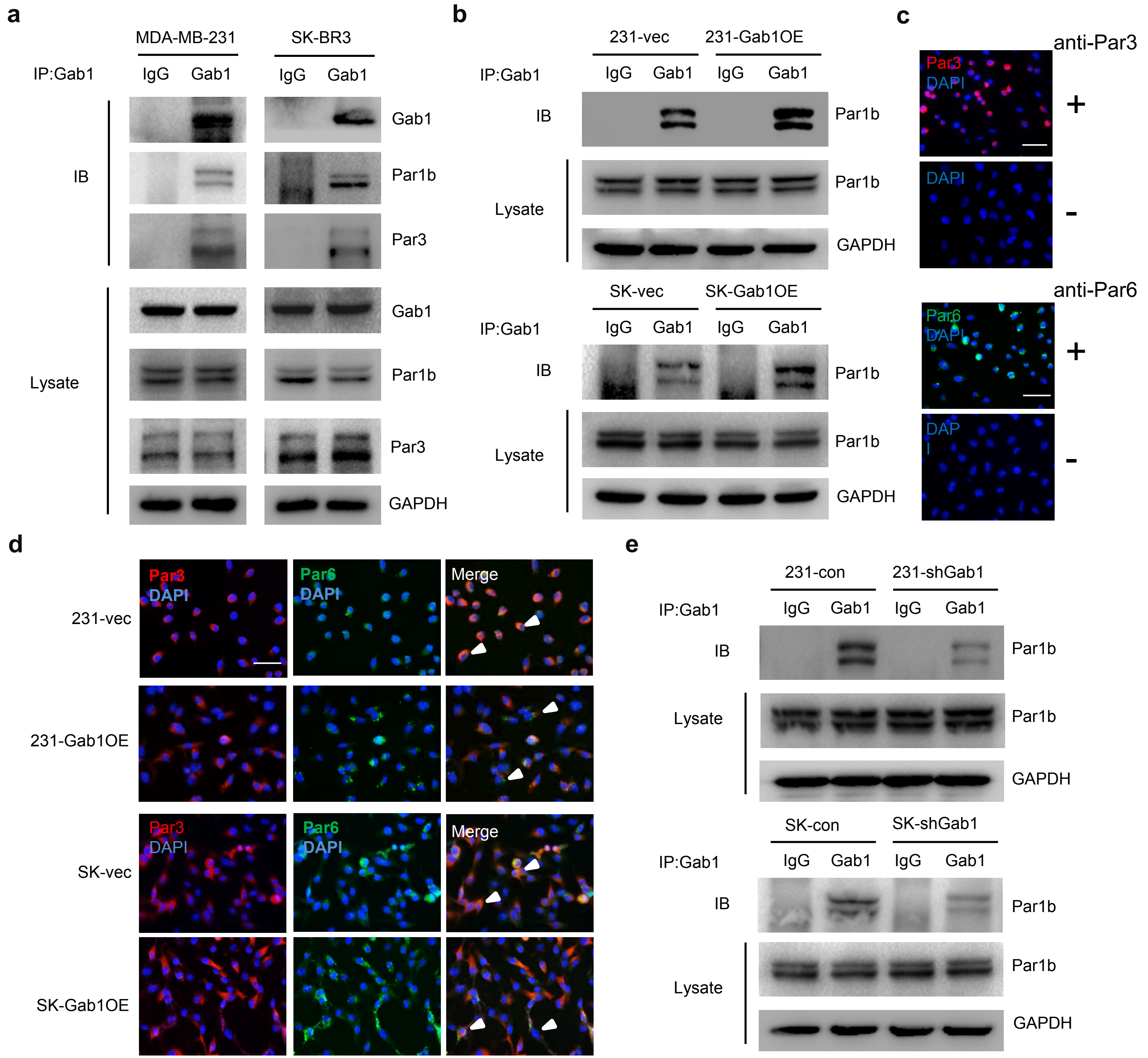
a
MDA-MB-231
SK-BR3
IP:Gab1
IgG
Gab1
IgG
Gab1
IB
Gab1
Par1b
Par3
Lysate
Gab1
Par1b
Par3
GAPDH
b
231-vec
231-Gab1OE
IP:Gab1
IgG
Gab1
IgG
Gab1
IB
Par1b
Lysate
Par1b
GAPDH
SK-vec
SK-Gab1OE
IP:Gab1
IgG
Gab1
IgG
Gab1
IB
Par1b
Lysate
Par1b
GAPDH
c
anti-Par3
Par3
DAPI
+
DAPI
-
anti-Par6
Par6
DAPI
+
DAP
I
-
d
231-vec
231-Gab1OE
SK-vec
SK-Gab1OE
Par3
DAPI
Par6
DAPI
Merge
Par3
DAPI
Par6
DAPI
Merge
e
231-con
231-shGab1
IP:Gab1
IgG
Gab1
IgG
Gab1
IB
Par1b
Lysate
Par1b
GAPDH
SK-con
SK-shGab1
IP:Gab1
IgG
Gab1
IgG
Gab1
IB
Par1b
Lysate
Par1b
GAPDH

Figure S3. Overexpression of Gab1 enhances while knockdown of Gab1 attenuates the interaction between Gab1 and Par1b. (a) Endogenous interaction of Gab1 with Par1b and Par3 is confirmed by co-IP assay in both MDA-MB-231-Gab1 and SK-BR3. (b) Stable overexpression of Gab1 enhances its interaction with Par1b in both MDA-MB-231 and SK-BR3. (c) Primary antibodies recognizing Par3 and Par6 used for IF staining are checked by no-primary antibody control assay. Scale Bar: 50μm (d) IF staining assay shows that the interaction of Par3 and Par6 is attenuated upon Gab1 overexpression. White arrow: typical cells with or without co-staining of Par3 and Par6 in the control or Gab1 overexpressed cells, Scale bars: 50μm (e) Knockdown of Gab1 attenuates its interaction with Par1b in both MDA-MB-231 and SK-BR3.

Figure S4

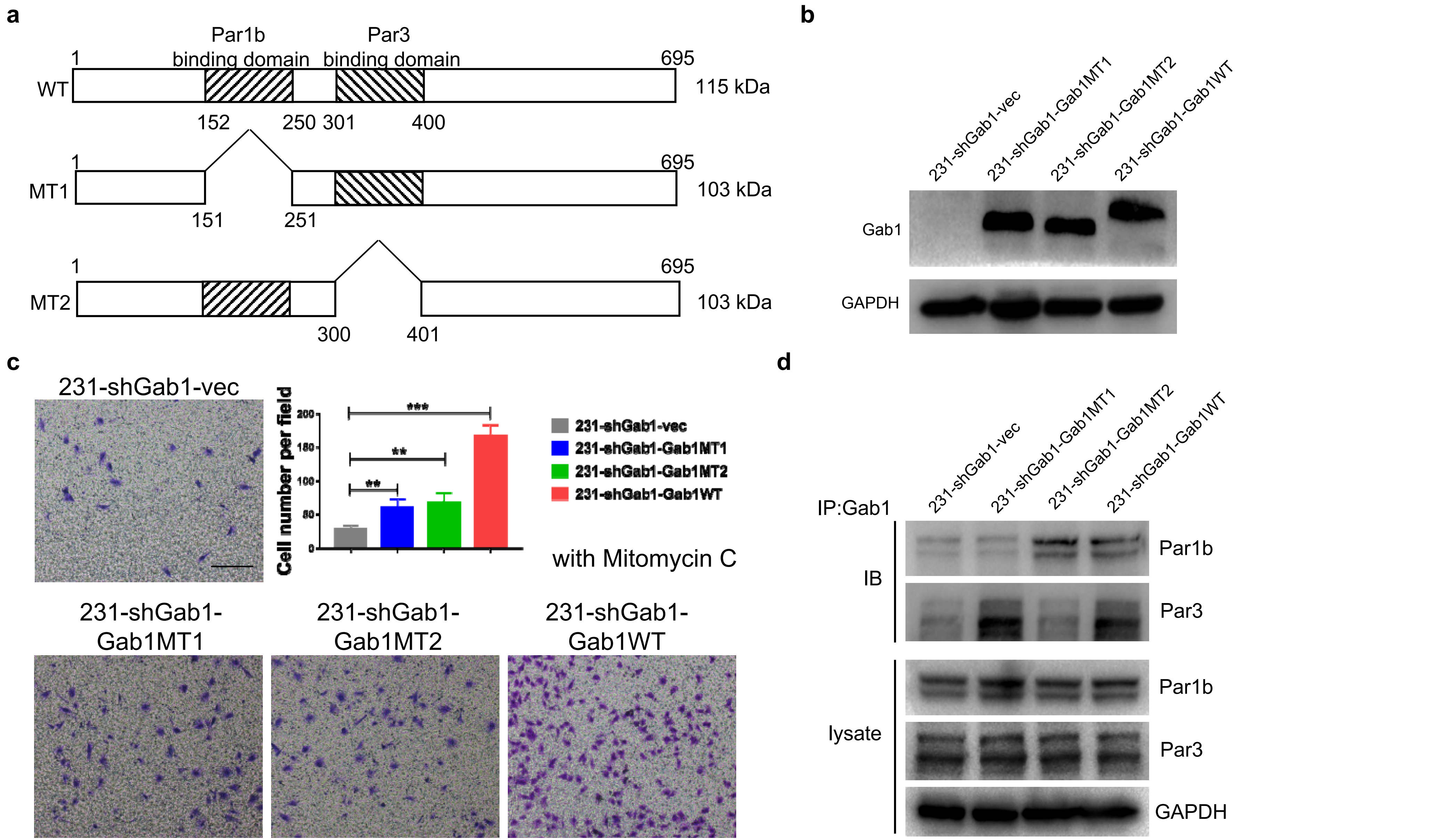

Figure S4. Deletion of Par1b or Par3 binding domain in Gab1 inhibits the interaction of Gab1 with Par1b or Par3. (a) Structure schematic of Par1b or Par3 binding domain deletion mutant Gab1. (b) Expression of wild type or mutant Gab1 is detected by western blot assay. (c) Cell migration is restored by re-expression of a fully functional but not a mutant Gab1 after treatment with Mitomycin C (10 μ g/ml) for 1 h. (d) Par1b or Par3 binding domain deficiency inhibits the interaction of Gab1 with Par1b or Par3 on the background of Gab1 knockdown. Scale bars: 200 μ m, Data are presented as means ± SEM. ＊＊: $p < 0.01$, ＊＊＊: $p < 0.001$

Figure S5

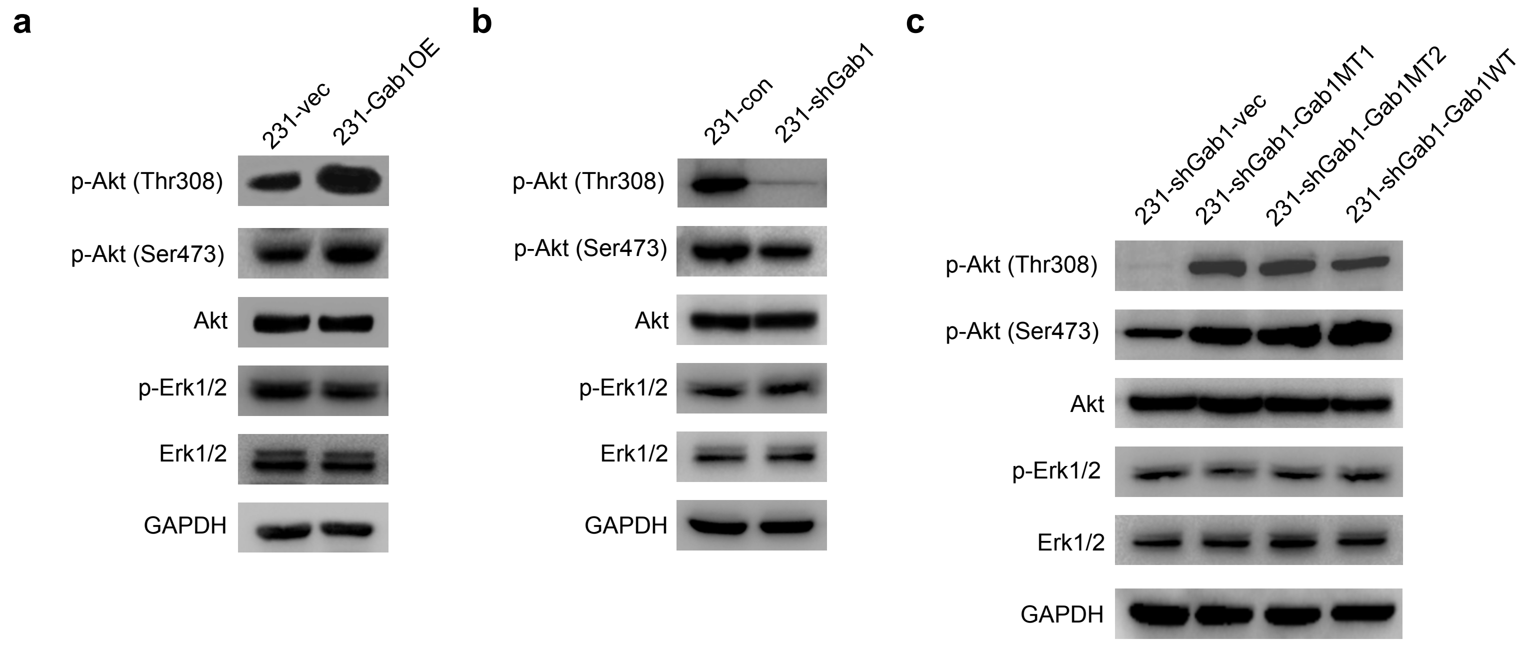

Figure S5. Expression of phosphorylated Akt and Erk after Gab1 overexpression or knockdown. (a) Gab1 overexpression enhances phosphorylation of Akt at Thr308 but not at Ser473 site, while it causes no influence on the phosphorylation of Erk1/2 in MDA-MB-231 cells. (b) Knockdown of Gab1 inhibits phosphorylation of Akt at Thr308 but not at Ser473 site, while it causes no influence on the phosphorylation of Erk1/2 in MDA-MB-231 cells. (c) Re-expression of a fully functional Gab1 but not a Par1b/Par3 binding domain deletion mutant Gab1 activates the phosphorylation of Akt, while either fully functional or mutant Gab1 has no effect on the phosphorylation of Erk1/2.

Figure S6

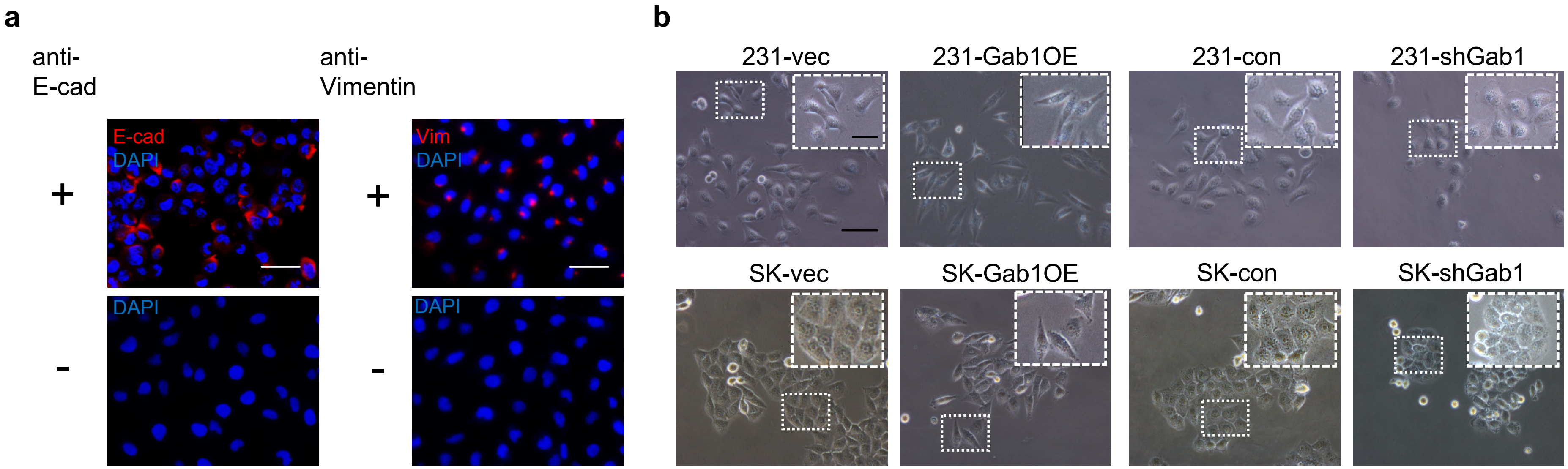

Figure S6. Overexpression of Gab1 induces EMT in BCa. (a) Primary antibodies against E-cad and Vimentin are checked by no-primary antibody control assay. Scale Bar: 50 μ m (b) Overexpression of Gab1 induces a mesenchymal-like morphological change and knockdown of Gab1 induces an epithelial-like morphological change in BCa cells. Scale Bar: 100 μ m for lower magnification; 50 μ m for higher magnification (up-right)

Figure S7

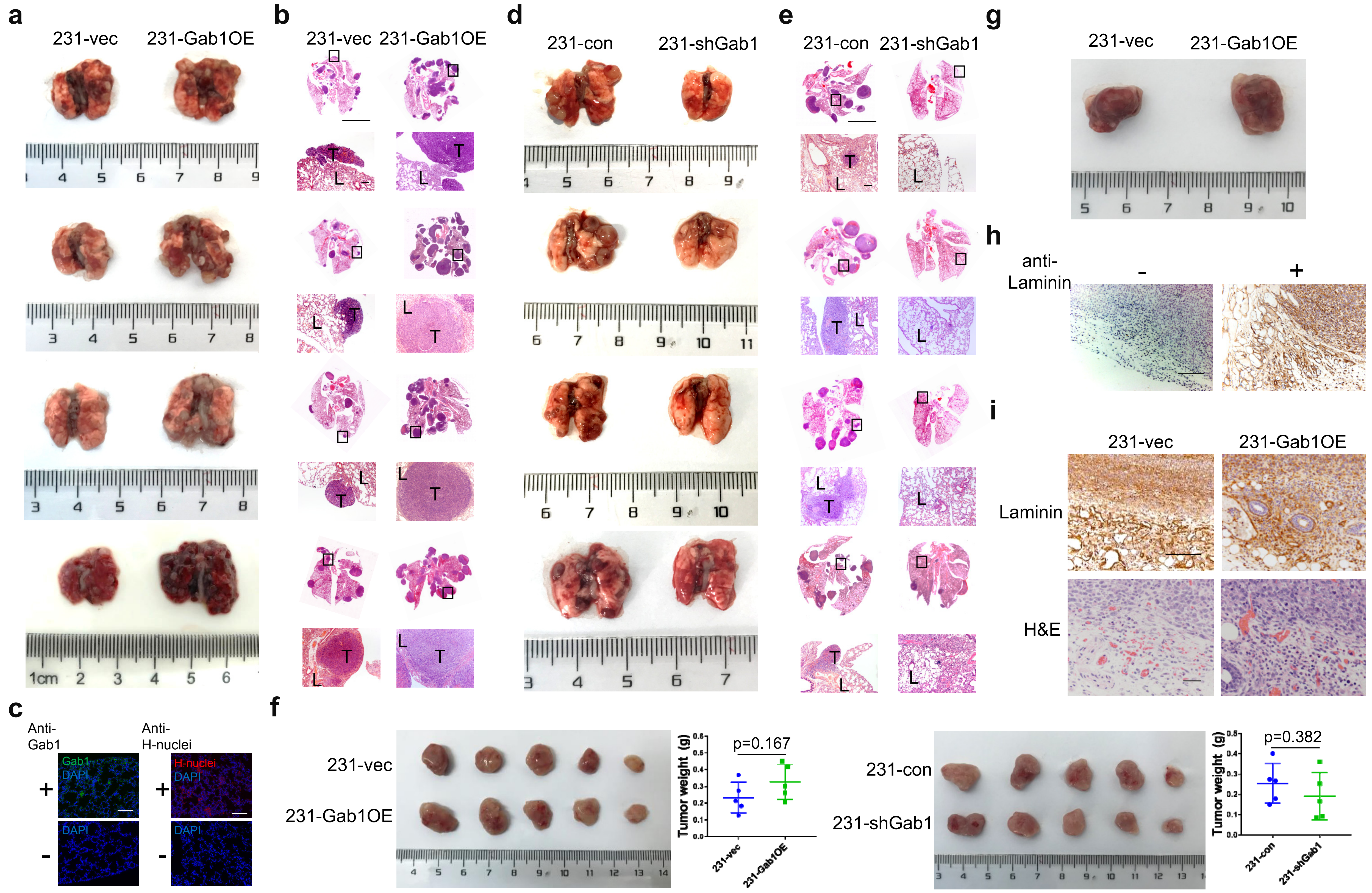

Figure S7. Upregulation of Gab1 promotes BCa metastasis *in vivo*. (a) Overexpression of Gab1 increases the number of metastatic nodes in lung. (b) Histopathological assessment in lung tissues from 231-vec or 231-Gab1OE group by H&E staining. (c) Primary antibodies against Gab1 and H-nuclei are checked by no-primary antibody control assay in mouse lung tissues. (d) Knockdown of Gab1 decreases the number of metastatic nodes in lung. (e) Histopathological assessment in lung tissues from 231-con or 231-shGab1 group by H&E staining. (f) Overexpression or knockdown of Gab1 does not display a significant influence on tumor growth *in vivo* by subcutaneous xenograft models. (g) The orthotopic tumor growth is similar between 231-vec and 231-Gab1OE group by orthotopic xenograft models. (h) Primary antibody against Laminin is checked by no-primary antibody control assay in mouse lung tissues. (i) The micro-invasion into adjacent normal tissues is enhanced in 231-Gab1OE group. Scale Bar: 1 cm for whole tissue scanning and 100 μ m for micrograph in (b) and (e), Scale Bar: 100 μ m for IF staining in (c) and for IHC staining in (h) and (i), H-nuclei: human nuclei, L: lung, T: tumor
