# Supplementary material for: Elevated expression of Gab1 promotes breast cancer metastasis by dissociating the PAR complex
Source: J Exp Clin Cancer Res. 2019 Jan 21;38:27. doi: 10.1186/s13046-019-1025-2 (PMC6341703; doi:10.1186/s13046-019-1025-2)
Supplement: Supplementary file 3 — Table S1. Clinical data from patients for western blot assay (Fig. S1a). Table S2. Clinical data from BCa patients and benign mammary hyperplastic controls for IHC staining (Fig. 1c). Table S3. Clinical data from BCa patients and benign mammary hyperplastic controls for IF staining (Figure S1d). Table S4. Clinical data from patients with metastatic or primary BCa for IHC staining (Fig. 1d). (PDF 77 kb) [file 13046_2019_1025_MOESM3_ESM.pdf]

Additional file 3:

Table S1: Clinical data from patients for western blot assay (Fig. S1a).

Patient Number	Age	Tumor type	TNM stage
P1	65	right invasive ductal carcinoma	T2N0M0
P2	70	left invasive ductal carcinoma	T2N0M0
P3	68	right invasive ductal carcinoma	T2N0M0
P4	52	right invasive ductal carcinoma	T2N0M0
P5	62	left invasive ductal carcinoma	T2N3M0
P6	73	right invasive ductal carcinoma	T2N0M0
P7	56	left invasive ductal carcinoma	T2N1M0
P8	48	left invasive ductal carcinoma	T1N0M0

P: patient

Table S2: Clinical data from BCa patients and benign mammary hyperplastic controls for IHC staining (Fig. 1c).

No.	Molecular subtype	ER	PR	HER2	Age	Tumor type	TNM stage	Metastasis
1	control	—	—	—	51	—	—	—
2	control	—	—	—	35	—	—	—
3	control	—	—	—	47	—	—	—
4	control	—	—	—	37	—	—	—
5	control	—	—	—	45	—	—	—
6	control	—	—	—	69	—	—	—
7	Luminal	+	+	-	59	right invasive ductal carcinoma	T2N2M0	No
8	Luminal	+	+	-	59	right invasive ductal carcinoma	T2N1M0	No
9	Luminal	+	+	-	43	left invasive ductal carcinoma	T1N0M0	No
10	Luminal	+	+	-	41	left invasive ductal carcinoma	T1N0M0	No
11	Luminal	+	+	-	45	right invasive ductal carcinoma	T2N1M0	No
12	Luminal	+	+	-	50	right invasive ductal carcinoma	T1N0M0	No
13	HER2	-	-	+	—	left invasive ductal carcinoma	T2N2M0	No
14	HER2	-	-	+	55	right invasive ductal carcinoma	T2N0M0	No
15	HER2	-	-	+	57	left invasive ductal carcinoma	T2N0M0	No
16	HER2	-	-	+	63	right invasive ductal carcinoma	T2N0M0	No
17	HER2	-	-	+	53	right invasive ductal carcinoma	T2N2M0	No
18	HER2	-	-	+	62	right invasive ductal carcinoma	T2N1M0	No
19	TNBC	-	-	-	72	left invasive ductal carcinoma	T2N0M0	No
20	TNBC	-	-	-	45	left invasive ductal carcinoma	T1N0M0	No
21	TNBC	-	-	-	44	right invasive ductal carcinoma	T1N0M0	No
22	TNBC	-	-	-	53	right invasive ductal carcinoma	T2N0M0	No
23	TNBC	-	-	-	56	left invasive ductal carcinoma	T2N0M0	No
24	TNBC	-	-	-	47	left invasive ductal carcinoma	T2N0M0	No

+: positive; -: negative; —: Data not detected;

ER: estrogen receptor; PR: progesterone receptor; HER2: human epidermal growth factor receptor 2; TNBC: triple negative breast cancer;

No-primary antibody control assay for Gab1 IHC staining is carried out using tissue sections from patient labeled in blue.

Typical image for Gab1 IHC staining (shown in Fig. 1c) is captured using tissue sections from patients labeled in red.

Table S3: Clinical data from BCa patients and 6 benign mammary hyperplastic controls for IF staining (Fig. S1d).

No.	Molecular subtype	ER	PR	HER2	Age	Tumor type	TNM stage	Metastasis
1	control	—	—	—	34	—	—	No
2	control	—	—	—	39	—	—	No
3	control	—	—	—	44	—	—	No
4	control	—	—	—	47	—	—	No
5	control	—	—	—	49	—	—	No
6	control	—	—	—	41	—	—	No
7	Luminal	+	+	-	73	invasive ductal carcinoma	T1N0M0	No
8	Luminal	+	+	-	45	invasive ductal carcinoma	T1N1M0	No
9	Luminal	+	+	-	55	invasive ductal carcinoma	T1N0M0	No
10	Luminal	+	+	-	70	invasive ductal carcinoma	T1N0M0	No
11	Luminal	+	+	-	56	invasive ductal carcinoma	T1N0M0	No
12	Luminal	+	+	-	50	invasive ductal carcinoma	T2N2M0	No
13	HER2	-	-	+	36	invasive ductal carcinoma	T2N2M0	No
14	HER2	-	-	+	41	invasive ductal carcinoma	T1N0M0	No
15	HER2	-	-	+	63	invasive ductal carcinoma	T2N0M0	No
16	HER2	-	-	+	68	invasive ductal carcinoma	T1N1M0	No
17	HER2	-	-	+	54	invasive ductal carcinoma	T2N2M0	No
18	HER2	-	-	+	59	invasive ductal carcinoma	T1N0M0	No
19	TNBC	-	-	-	51	invasive ductal carcinoma	T2N0M0	No
20	TNBC	-	-	-	43	invasive ductal carcinoma	T1N0M0	No
21	TNBC	-	-	-	79	invasive ductal carcinoma	T1N0M0	No
22	TNBC	-	-	-	45	invasive ductal carcinoma	T1N0M0	No
23	TNBC	-	-	-	59	invasive ductal carcinoma	T1N0M0	No
24	TNBC	-	-	-	65	invasive ductal carcinoma	T2N1M0	No

+: positive; -: negative; —: Data not detected;

ER: estrogen receptor; PR: progesterone receptor; HER2: human epidermal growth factor receptor 2; TNBC: triple negative breast cancer;

No-primary antibody control assay for EpCAM (No.1) and Gab1 (No. 7) IF staining is carried out using tissue sections from patients labeled in blue.

Typical image for EpCAM-Gab1 IF co-staining (shown in Fig. S1d) is captured using tissue sections from patients labeled in red.

Table S4: Clinical data from patients with metastatic or primary BCa for IHC staining (Fig. 1d).

	Molecular subtype	ER	PR	HER2	Age	Tumor type	TNM stage	Metastasis
P1	HER2	-	-	+	61	left invasive ductal carcinoma	T2N3M0	No
P2	HER2	-	-	+	55	right invasive ductal carcinoma	T1N1M0	No
P3	HER2	-	-	+	49	left invasive ductal carcinoma	T1N2M1	Yes
P4	HER2	-	-	+	73	left invasive ductal carcinoma	T2N2M1	Yes
P5	TNBC	-	-	-	78	left invasive ductal carcinoma	T2N0M0	No
P6	TNBC	-	-	-	63	right invasive ductal carcinoma	T2N0M1	Yes

P: patient; +: positive; -: negative;

ER: estrogen receptor; PR: progesterone receptor; HER2: human epidermal growth factor receptor 2; TNBC: triple negative breast cancer
