# Supplementary material for: Elevated expression of Gab1 promotes breast cancer metastasis by dissociating the PAR complex
Source: J Exp Clin Cancer Res. 2019 Jan 21;38:27. doi: 10.1186/s13046-019-1025-2 (PMC6341703; doi:10.1186/s13046-019-1025-2)
Supplement: Supplementary file 4 — Supplementary methods. (PDF 70 kb) [file 13046_2019_1025_MOESM4_ESM.pdf]

Additional file 4:

Supplementary methods

Cell proliferation

To evaluate the role of Gab1 in BCa cell proliferation, 2.5×10^3 cells per well were seeded in a 96-well plate and were incubated for 6 days. For CCK-8 (Dojindo, Kumamoto, Japan) assay, cells were incubated with CCK-8 for 2 hrs at 37°C with 5% CO_2 and then the absorbance values were measured at 450 nm using BioTek Synergy HT microplate reader.

In vivo subcutaneous xenografts assay

For subcutaneous xenograft assay, a total of 2×10^6 cells were suspended in 50 μl basic DMEM medium and mixed with 50 μl matrigel (BD Biosciences, CA, USA). The mixtures were injected subcutaneously into the flank of mouse leg in six-week-old female nude mice (n=5 for each group). Three weeks after inoculation, mice were sacrificed and xenografts were collected for photographs and weighing.

Orthotopic mammary xenografts assay

For orthotopic mammary xenografts assay, 2×10^6 cells were suspended in 50 μl basic DMEM medium and mixed with 50 μl matrigel. Total 100 μl mixtures were orthotopically injected into the 4th mammary fat pad of nude mice. Ten weeks later, mice were sacrificed and then orthotopic tumors mass and respective lungs were collected for photographs, and tissue sections were prepared for H&E staining and Laminin IHC staining, respectively.
